# Coiled-coil heterodimer-based recruitment of an exonuclease to CRISPR/Cas for enhanced gene editing

**DOI:** 10.1038/s41467-022-31386-1

**Published:** 2022-06-23

**Authors:** Duško Lainšček, Vida Forstnerič, Veronika Mikolič, Špela Malenšek, Peter Pečan, Mojca Benčina, Matjaž Sever, Helena Podgornik, Roman Jerala

**Affiliations:** 1 Department of Synthetic Biology and Immunology, National Institute of Chemistry, Hajdrihova 19, Ljubljana, 1000 Slovenia; 2 EN-FIST Centre of Excellence, Trg Osvobodilne fronte 13, Ljubljana, 1000 Slovenia; 3 Department of Hematology, Division of Internal Medicine, University Medical Centre Ljubljana, Zaloška 7, Ljubljana, 1000 Slovenia; 4 Graduate School of Biomedicine, University of Ljubljana, Ljubljana, 1000 Slovenia; 5 Faculty of Medicine, University of Ljubljana, Korytkova 2, Ljubljana, 1000 Slovenia; 6 Faculty of Pharmacy, University of Ljubljana, Aškerčeva cesta 7, Ljubljana, 1000 Slovenia

**Keywords:** Synthetic biology, CRISPR-Cas9 genome editing, Double-strand DNA breaks, Non-homologous-end joining

## Abstract

The CRISPR/Cas system has emerged as a powerful and versatile genome engineering tool, revolutionizing biological and biomedical sciences, where an improvement of efficiency could have a strong impact. Here we present a strategy to enhance gene editing based on the concerted action of Cas9 and an exonuclease. Non-covalent recruitment of exonuclease to Cas9/gRNA complex via genetically encoded coiled-coil based domains, termed CCExo, recruited the exonuclease to the cleavage site and robustly increased gene knock-out due to progressive DNA strand recession at the cleavage site, causing decreased re-ligation of the nonedited DNA. CCExo exhibited increased deletion size and enhanced gene inactivation efficiency in the context of several DNA targets, gRNA selection, Cas variants, tested cell lines and type of delivery. Targeting a sequence-specific oncogenic chromosomal translocation using CCExo in cells of chronic myelogenous leukemia patients and in an animal model led to the reduction or elimination of cancer, establishing it as a highly specific tool for treating CML and potentially other appropriate diseases with genetic etiology.

## Abstract (web summary)

The CRISPR/Cas system has emerged as a powerful and versatile genome engineering tool. Here the authors couple Cas9 to effector protein Exonuclease III via coiled-coil mediated interactions, termed CCExo, leading to increased deletion sizes and enhanced gene knock-out efficiencies in cell lines, primary cells and in vivo.

## Introduction

The bacterial and archaeal-derived CRISPR/Cas gene editing system allows guide RNA (gRNA)-guided site-specific cleavage of DNA^1^ and has been widely used in different areas of biological research, such as development of cell lines and animal models and other areas such as drug, food and fuel production^2,3^. CRISPR/Cas also holds considerable potential for treatment of various genetic disorders through either correcting mutations driving disease pathogenesis or by disrupting the disease-causing genes^4^, thus making CRISPR/Cas an indispensable tool for translational medicine^5^. Therefore, improvements of the efficiency of CRISPR are eagerly adopted in biotechnological and biomedical applications. The ribonucleoprotein (RNP) complex is guided via specific gRNA to the targeted genomic region where the catalytic activity of the Cas protein results in a double stranded break (DSB)^3,6^, which is subjected to cellular repair mechanisms, mainly non-homologous end joining (NHEJ) and homology-directed repair (HDR)^7^. These cell-repair mechanism may be exploited for implementation of desired genomic rearrangements such as “knock-out” or “knock-in” mutations. The most frequent mode of repair within a cell is error-prone NHEJ, which often results in lesions of several nucleotides^8^ leading to deletions or frameshift mutations, which are manifested in non-functional transcripts of targeted genes^9^.

Several modifications have been implemented to enhance efficiency or tailor certain aspects of the CRISPR/Cas system such as optimization of gRNA design and chemical modifications^10–12^, Cas mutants and orthologues including Cas9 nickase^13^, catalytically inactive Cas9 (dCas9) fused with FokI endonuclease^12^, high fidelity variants^14,15^, different CRISPR type Cas proteins, such as Cas3^16^, Cas13^17^ and Cas12a^18^, fusion to other functional domains or moieties, such as FokI endonuclease^12^, reverse transcriptase^19^, cytidine deaminase^20^, Rad52^21^ and chemical^22^ or light^22^ inducible domains or to intein domains^23^, to allow tight spatio-temporal regulation of Cas9 catalytic activity.

Exonucleases have been used in combination with DNA editing enzymes. Exonucleases (Exo) catalyze the excision of nucleoside monophosphates from the 3’ or 5’ DNA termini^24–26^. Co-expression of 3ʹ repair exonuclease 2 (Trex2) with the homing endonuclease I-SceI or codelivery of 3’−5’ exonuclease ExoI and TALENs has been shown to enhance targeted gene editing^27,28^. Other recent reports show co-expression of the CRISPR/Cas system with ExoI^29^ or fusion with T5 exonuclease^30^, resulted in an increase of indel frequencies, albeit to a low extent.

While diverse genetic fusions have substantially expanded the potential of CRISPR/Cas applications, potential drawbacks include possible fusion protein folding issues and the size of fusion proteins that may exceed the size limit for the viral vector. An alternative to genetic fusion, which may circumvent these issues and enable co-delivery by viral vectors, could be the use of coiled-coil dimerization (CC) peptides. These small peptides can achieve tight and specific homo- or heterodimerization pairing based on the combination of designable electrostatic and hydrophobic interactions^31^. CCs have been used as modular protein building blocks^32–34^ as well as to combine within cells different protein functional moieties, such as dCas9 and transcriptional regulatory domains or protease-based signaling pathways^35,36^.

Here, we report the design and application of Cas proteins of different CRISPR type systems to which exonucleases are recruited via coiled-coil peptides to increase the efficiency of gene editing, further referred to as CCExo (**C**RISPR-**C**oiled-coil–**Exo**nuclease). Recruitment of exonucleases to the Cas9 or Cas12a protein was achieved via peptides of different pairs of CCs genetically fused to each enzyme. CC-based recruitment was superior to coexpression and direct fusion, wherein the highest mutation frequency and deletion length was observed for the strongest affinity CC pair. Coupling of Cas9 and exonuclease resulted in a robust improvement of gene inactivation for multiple DNA targets in several cell-lines and primary cells. The application of CCExo system was demonstrated in primary cells of patients with chronic myeloid leukemia (CML) harboring the *BCR-ABL* fusion gene and in a xenograft animal model, where cancer suppression and elimination was induced via plasmid DNA or RNP delivery, thus underlining the CCExo approach as a potential therapeutic option for treatment of CML and other disorders with genetic etiology.

## Results

### Co-expression of CRISPR/Cas with exonucleases increases indel mutation frequency

**Fig. 1:**
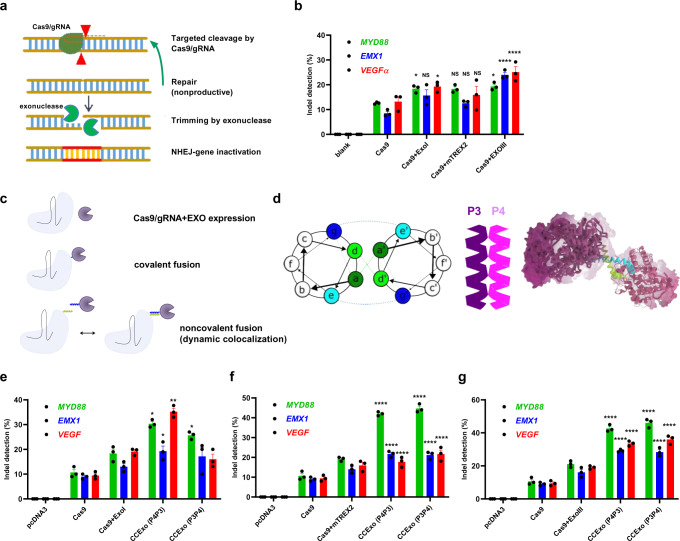
Tethering of Cas9 to exonucleases via coiled-coil dimer peptides for the enhanced efficiency of genome editing. Schematic presentation of exonucleases on excessive DNA trimming for enhanced gene inactivation (**a**). HEK293 cells (2*10^5^ cells/ml) were co-transfected with plasmids encoding Cas9, gRNA and an exonuclease (human ExoI, mouse TREX2 or *E.coli* ExoIII) (600 ng of each component). 48 h later genomic DNA was isolated and T7E1 assay for indel detection was performed. Data present three separate experiments (*n* = 3). *P < 0.05, ****P < 0.0001. All *P* values are from ordinary one-way ANOVA followed by Tukey’s multiple comparisons test to Cas9 values. Data are presented as mean values ± SEM as appropriate (**b**). Different approaches for achieving increased gene editing using Cas9 and exonucleases (**c**). Schematic presentation of P3 and P4 parallel coiled-coil dimer interactions tethering Cas9 and exonuclease with green and blue letters presenting hydrophobic and charged interacting amino acid residues, respectively (**d**). HEK293 cells (2*10^5^ cells/ml) were transfected with plasmids encoding gRNA and CCExo (Cas9-P3 or -P4, P3- or P4-ExoI, P3 or P4-mTREX2 or P3- or P4-ExoIII) (600 ng of each component). 48 h later genomic DNA was isolated and T7E1 assay was performed to assess indel frequency. Indel frequencies were compared between editing with Cas9 alone, co-expression of Cas9 with an exonuclease or coiled-coil based coupling of Cas9 to an exonuclease, wherein different fusion variants of P3 and P4 coils were tested (P4P3 denotes transfection with Cas9-P4 coexpressed with P3-Exo- and P3P4 denotes Cas9-P3 coexpressed with P4-Exo). Indel frequencies were tested for human EXOI (**e**), mouse TREX2 (**f**) and *E.coli* ExoIII (**g**). Data present results from three separate experiments (*n* = 3). *P < 0.05, **< 0.01, ***< 0.001 and ****P < 0.0001. All *P* values are from ordinary one-way ANOVA followed by Tukey’s multiple comparisons test compared to Cas9 values. Data are presented as mean values ± SEM as appropriate.

Formation of DSBs triggers recruitment of the NHEJ machinery, which includes proteins that sense DNA breakage and DNA modifying enzymes, to the break point^8^. NHEJ may also repair the target DNA without modifications and Cas9 may have to cleave the same target site multiple times, until the target DNA no longer contains the Cas9/gRNA recognition site. We reasoned that in order to increase gene disruption efficiency, DNA end-processing enzymes, such as exonucleases may be able to generate DNA recessions that prevent ligation of intact target DNA (Fig. 1a). We analyzed the effect of co-expression of Cas9 with five exonucleases of different origin with 3’−5’ or 5’−3’ activity on the frequency of indel formation. The HEK293 reporter cell line stably expressing eGFP was transfected with a plasmid encoding the CRISPR/Cas9 system targeting the *eGFP* gene and monitored for indel formation through fluorescence decrease. We observed a 40% decrease of fluorescence in CRISPR/Cas9 treated cells relative to control cells, and a further decrease in cells co-transfected with plasmids encoding human ExoI, *E.coli* ExoIII or mouse TREX2, while human FEN1 and WRN exonucleases had no significant effect (Supplementary Fig. 1). ExoI, ExoIII and TREX2 exonucleases were further analyzed in combination with CRISPR/Cas9 targeting three human genomic targets, *MYD88*, *VEGFα* and *EMX1* (Fig. 1b). Although we observed an increase in the indel mutation frequency in all cases of exonuclease co-expression, co-expression of ExoIII most potently increased the occurrence of indels (Fig. 1b).

We hypothesized that the proximity of Cas9 and the exonuclease could be synergistic for the formation of in situ genetic lesions and to this effect we constructed genetic fusions of ExoIII to the N- or C- terminus Cas9 connected via a 10 or 20 amino-acid (aa) (GlySer) linker. Surprisingly however, only the N-terminal fusion variant of Cas9 and ExoIII, connected via a 20 aa linker, resulted in editing of genomic DNA, and even in this case with an efficiency comparable to co-expression of the two protein components (Supplementary Fig. 2a, b).

### Recruitment of ExoIII to Cas9 via coiled-coil dimerization improves indel formation efficiency

Since covalent fusion of Cas9 with ExoIII did not increase the efficiency of indel formation, we hypothesized that this might due to structural constraints that might prevent efficient DNA processing or alternatively that it could be due to impaired protein folding. We reasoned that recruitment of the exonuclease to the location of DSB should nonetheless be beneficial. Coiled-coil peptides can be used to bring the desired protein subunits in proximity in a non-covalent manner. For this purpose, Cas9 and ExoIII were each genetically fused to previously designed four-heptad repeat coiled-coil dimer forming peptides, P3 and P4, respectively, that form heterodimers based on an interplay of hydrophobic and electrostatic interactions^33^ (Fig. 1c–d). HEK293 cells were transfected with plasmids encoding wild type (wt) or coiled-coil-fused Cas9 and ExoI, TREX2 or ExoIII. We found that coiled-coil driven dimerization of Cas9 significantly increased genome editing with all tested exonucleases, compared to the CRISPR/Cas system for all tested genomic targets (Fig. 1e–g). While the effect of tested exonucleases seemed to depend somewhat on the specific genomic context, ExoIII induced the highest indel frequencies for all targeted regions (Fig. 1g). Interestingly, not only indel occurrence frequency, but also the length of deletions increased in CCExo mediated *MYD88* gene editing, where we observed up to 40 nt deletions (Supplementary Fig. 3), determined by NGS analysis of PCR amplicons, surrounding the gRNA targeting site. Enhanced genome editing due to CCExo was also observed using online tools for Sanger sequences analysis for *CASP3* gene disruption in transfected HEK293 cells, with a 7-fold increase in editing efficiency compared to the basic CRISPR system (Supplementary Fig. 4).

In order to assess the possible effect on off-target sites^5,37^, three predicted off-target sites (*ANKRD52*, *FUT9* and *PSKH2*) for *MYD88* targeting gRNA were tested. No undesired DNA cleavage was observed at any of the analyzed non-target genomic sites (Supplementary Fig. 5), whereas on-target activity was determined (Fig. 1f).

### CCExo reduces DNA religation in situ

The underlying hypothesis for our strategy was that the proximity of ExoIII to the Cas9 cleavage site triggers formation of genetic lesions and prevents scar-less repair through NHEJ. In order to test this mechanism we performed an in vitro digestion assay in the presence of a DNA ligase with purified recombinant Cas9^38^ and ExoIII proteins and their variants (Supplementary Fig. 6). Their synergistic action was tested in vitro for cleavage of DNA templates containing appropriate gRNA targets (Supplementary Fig. 7a) with the addition of a DNA ligase into the cleavage reaction. Targeted cleavage of DNA was performed using RNPs. After incubation time, heat inactivation of RNPs was performed. Then, re-ligation was achieved via T4 DNA ligase mediated action. Re-ligation of the template DNA was observed in Cas9, Cas9-N5 or Cas9+ExoIII treatment, but was abolished in the case of CCExo initiated DNA cleavage. This suggests that ExoIII recruitment to Cas9 via CCs results in a rapid generation of DNA recessions, for which we assume that scar-less re-ligation of the processed DNA target molecules is prevented (Supplementary Fig. 7b, c). Based on agarose gel analysis we observed formation of a linearized form of plasmid DNA after targeted DNA cleavage with the same mobility as the EcoRI digested positive control. The intensity of the linearized form decreased with the addition of a ligase in the case of EcoRI and Cas9 but not in case of CCExo treatment. We also observed two additional DNA bands (even in the blank undigested sample), which we assume are relaxed-coiled and supercoiled forms of plasmid DNA^39^. Interestingly, the addition of N6-ExoIII to the reaction with Cas9-N5 demonstrated a strong increase of plasmid linearization, in contrast to the addition of ExoIII without the coiled-coil domain. It is known that Cas9 dissociates very slowly from substrate DNA post cleavage^40,41^. A likely explanation for the augmented dsDNA cleavage via CCExo may be that the ExoIII at the Cas9 cleavage site progressively digests the cleaved DNA from the 3’ end which could trigger faster dissociation of Cas9 from the DNA, freeing it for acting on additional target sites. By using RNPs containing Cas9-N5, we determined that increased linearization is not due to enhanced cleavage efficiency of Cas9-N5 as the results of Cas9-N5 mediated DNA digestion were similar to Cas9 directed DNA template cleavage. The intensity of the linearized plasmid band is increased with increased N6-ExoIII concentration, whereas the sum of supercoiled form of the DNA substrate was diminished (Supplementary Fig. 7d, e), which was not observed when ExoIII, lacking CC segment was added. Greater spatial proximity of ExoIII and Cas9 due to coiled-coil mediated association might enhance Cas9 dissociation from the DNA substrate, although this mechanism would need further investigation. In conlusion, we observed, as with plasmid-based assays, that CCExo more efficiently prevents religation of DNA in comparison to Cas9, Cas9-N5 or Cas9 combined with ExoIII.

### Efficiency of CCExo in different cell types and in vivo

In order to assess the robustness of the CCExo system, we analyzed its efficiency in different cell lines. gRNA targeting the murine *Ptgs1* gene led to a significant increase in genome modification in the mouse Neuro2A cell line (Supplementary Fig. 8), where ExoIII tethering to Cas9 via CCs demonstrated better efficiency than TREX2 or ExoI.

Further, an important aspect is the ability to modify somatic adult cells in vivo to assess the applicability to therapeutic targets linked to disease. CCExo coding plasmids were introduced via a hydrodynamic injection into mice^38,42^ with the analysis of indels in liver-derived cells. A 3-fold increase in the efficiency of indel formation in CCExo-treated mouse cells relative to the standard CRISPR system was observed (Supplementary Fig. 9a–b). To rule out enhanced genome editing of *Ptsg1* gene in mouse liver cells due to variable Cas9 expression levels we performed anti-Cas9 staining in permeabilized liver cell suspensions, obtained from harvested liver samples of treated animals. Liver cells from control, CAG-Cas9 mice, expressing Cas9^43^ and from negative Hsd-ICR mice were included as Cas9 staining positive and negative controls. No statistical difference in Cas9 protein, expressed from plasmids encoding Cas9 or CCExo was seen in treated animals, concluding that enhanced genome editing is not due to differential protein expression levels (Supplementary Fig. 9c–e). This demonstrated the efficiency of CCExo in vivo with hydrodynamic delivery as a suitable method for genomic editing in an animal model.

### Influence of coiled-coil peptide properties on the efficiency of CCexo

**Fig. 2:**
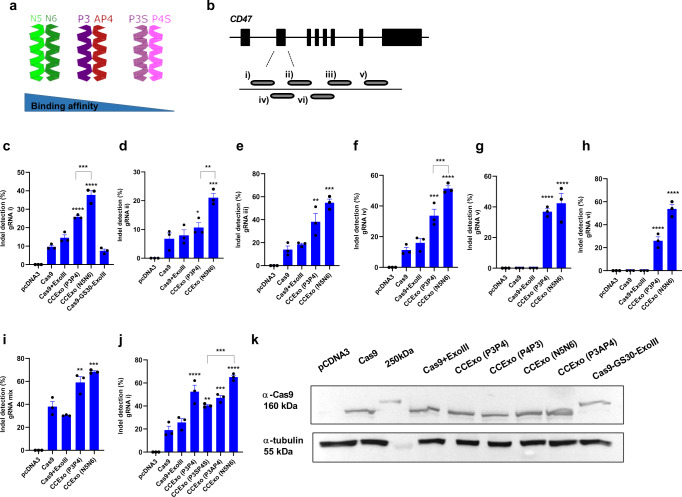
The effect of coiled-coil forming peptide affinity on the efficiency of CCExo. Schematic presentation of different parallel or anti-parallel coiled coil pairing. N5N6 (strong parallel CC pair), P3AP4 (anti-parallel CC pair) and P3SP4S (weak parallel CC pair) (**a**). Diagram showing gRNA positions within exon 2 of the human *CD47* gene (**b**). Indel mutation quantification by the T7E1 assay, determined 48 h post transfection (HEK293 cells (2*10^5^ cells/ml) were transfected with plasmids encoding gRNA and CCExo) for several tested gRNAs (**c**–**h**), a combination of all gRNAs (**i**), and gRNA1 in combination with CCExo containing coiled-coils with increasing affinity (P3P4 < N5N6) (**j**). Data present three individual separate experiments (*n* = 3). *P < 0.05, **< 0.01, ***< 0.001 and ****P < 0.0001. All *P* values are from ordinary one-way ANOVA followed by Tukey’s multiple comparisons test compared to Cas9 values or if stated otherwise by brackets. Data are presented as mean values ± SEM as appropriate (**c**–**j**). Expression of Cas9 or its variants in HEK293 cells 48 h post transfection determined by western blot and immunodetection using specific anti-Cas9 antibodies. α-tubulin was used as a loading control. A representative blot from two individual separate experiment is shown (**k**).

Designed CC dimers enable fine-tuning of the affinity as well as specification of the orientation (parallel or antiparallel) of interacting peptides^32,44^, which might affect the CCExo efficiency. We prepared constructs encoding Cas9 and ExoIII fused to several variants of parallel or antiparallel coiled-coil dimer-forming peptides from our collection of designed peptides^45,46^. In addition to the aforementioned P3-P4 pair, an N5-N6 pair^46^ with an increased coiled-coil forming affinity, as well as a P3S-P4S pair with weaker dimerization affinity were tested, along with a P3-AP4 pair that interacts in an antiparallel orientation (Fig. 2a). We designed gRNAs targeting the template and non-template DNA strand of the second exon of the human *CD47* gene (Fig. 2b). The highest affinity coiled-coil pair N5-N6 resulted in the highest gene editing frequency when each peptide was fused to Cas9 and ExoIII (Fig. 2c–i), respectively. To investigate if the observed effect resulted from the noncovalent coiled-coil-guided recruitment of ExoIII to Cas9 or if the effect could be replicated by a simple covalent linkage, an additional fusion variant of Cas9 and ExoIII was prepared, connected by an extended 30 aa linker to remove any steric hindrance and might enable enzymes to adopt a favorable relative orientation. This linker length mimics the length of coiled-coil peptides, to assess a possible role of coiled-coils functioning solely as a spacer. Gene editing ability of the covalent linker-mediated fusion of Cas9 and ExoIII resulted in similar indel frequency as Cas9 alone, demonstrating that the fusion protein is functional and that the superior performance results from the noncovalent coiled-coil based coupling in this context (Fig. 2c).

Importantly, in addition to the consistent improvement of the editing efficiency, recruitment of ExoIII to Cas9 via CC exhibited substantial gene editing in genomic target/gRNA combinations where no apparent editing resulted by application of the standard Cas9/gRNA system (Fig. 2g, h), indicating the increased robustness of CCexo with respect for the specific gRNA and genomic context. Enhanced genome editing using CCExo was also observed for multiple gRNAs against the same target gene (Fig. 2i). As stated above, N5-N6 tethering resulted in the highest indel efficiency, whereas using a weaker CC dimer-forming peptide pair P3S-P4S resulted in somewhat lower efficiency, nevertheless still higher compared to the standard CRISPR/Cas system (Fig. 2j). We found that the orientation (parallel-P3-P4 or antiparall-P3A-P4) of CC segments did not alter CCExo efficiency (Fig. 2j). These results suggest a mechanism of dynamic recruitment and release of the exonuclease at the DNA lesion, enabling DNA recession further from the Cas9 cleavage site independent of the precise relative orientation of the two enzyme domains.

Expression level of the wild type or coiled-coil-fused Cas9 was comparable, excluding the effect of different protein expression on measured indel frequencies (Fig. 2k). Decreased protein expression of CD47 due to gene disruption was confirmed by flow cytometry and reflected higher efficiency of the CCExo system in comparison to standard Cas9 editing either with a single gRNA or a combination of several gRNAs targeting exon 2 of the *CD47* gene (Supplementary Fig. 10). Improved gene editing via a strong pair of CC-forming peptides was also observed for the *ABL1* gene (Supplementary Fig. 11).

We next analyzed the kinetics of double-strand break generation in transfected cells through assessment of cleavage at several different time points, starting with 6 h post cell transfection. Indel detection via the T7E1 assay was detected at the first analyzed time point only in CCExo transfected cells, while no cleavage was detected in Cas9 or Cas9+ExoIII co-transfected samples. Furthermore, genome editing rate was higher at all-time points in CCExo transfected samples. We also generated a single plasmid, expressing Cas9-N5 and N6-ExoIII, split via a self-cleaving t2a peptide, enabling separate protein synthesis from one mRNA transcript^47^ presenting the possibility to use CCExo as a tool for therapeutic purposes, where all components could be incorporated into a single delivery system^48^. The single vector derived CCExo system, also showed increased genome modification with no statistical significant difference compared to the two-plasmid encoded CCExo system (Supplementary Fig. 12).

No difference in cell viability was observed for cell lines or primary cells transfected with plasmids encoding Cas9, ExoIII or any of the coiled-coil based fusions (Supplementary Fig. 13a, b), even if CCExo RNPs were electroporated into human T cells, obtained from healthy donors (Supplementary Fig. 13c).

Although no additional toxicity was reported when exonucleases were injected into animals or embryos^28,29^ we still carried out a substantial toxicity study on mice that were injected with recombinant CCExo components. We performed a complete comprehensive biochemical panel and pro- inflammatory cytokines determination in blood, drawn at various time points that resulted in no different outcomes when only Cas9 was injected, compared to CCExo protein injection or to PBS treated controls (Supplementary Fig. 14).

**Fig. 3:**
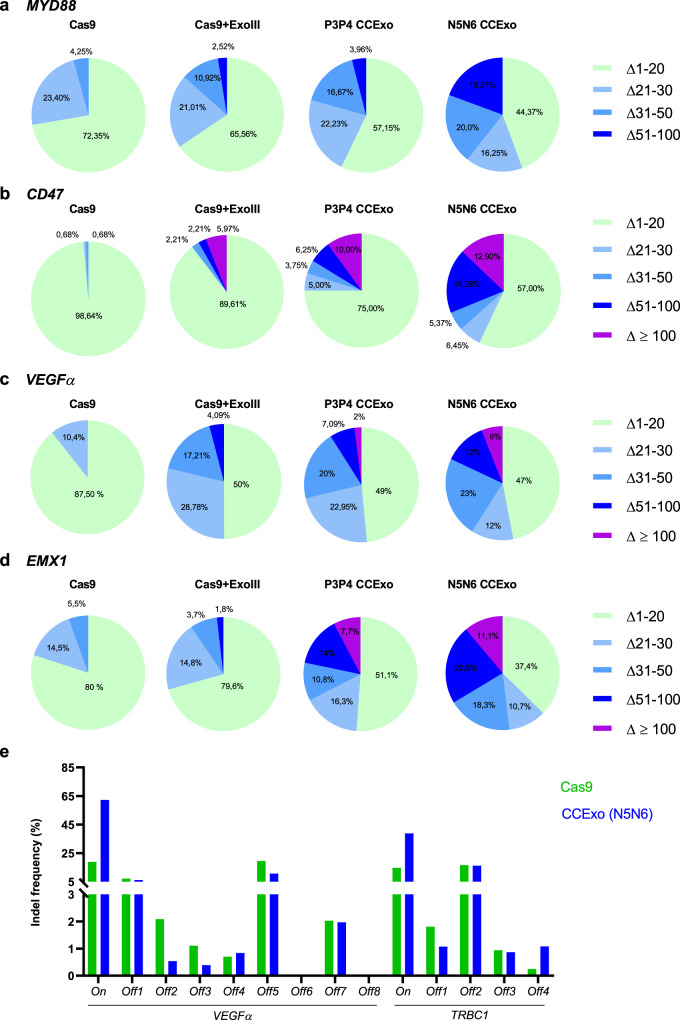
Coupling of exonuclease to Cas9 via CC increases the length of genomic deletions. HEK293 cells (2*10^5^ cells/ml) were transfected with plasmids encoding target gRNA and CCExo. Genomic DNA was isolated 48 h post transfection, the targeted region PCR amplified and subjected to NGS analysis. NGS analysis was performed for 200 bp amplicons for gRNA targeting *MYD88* gene (**a**) or 400 bp amplicons for gRNA targeting *CD47* gene (**b**), *VEGFα* gene (**c**) or *EMX1* gene (**d**). Diagrams are showing percentage (calculated based on number of total mutated reads) of deletions (Δ) of various lengths. Genome wide determinations of potential off-target activities of CCExo for *VEGFα* sites in HEK293 cells and for *TRBC1* site in human K562 cells, predicted by CIRCLE-seq (**e**).

To determine the influence of tethered ExoIII on genome integrity, PCR amplicons surrounding gRNA targeting sites were subjected to NGS analysis. We found that CCExo strongly augmented the length of deletions after the ExoIII DNA recession (Fig. 3a–d, Supplementary Fig. S15) which increases the probability of target gene inactivation. The percentage of deletions length (>50 bp) for *MYD88* or (~100 bp) for *CD47*, *VEGFα* and *EMX1* gene was increased by the CCExo system. The deletion length also depended on CC affinity used for tethering. Deletions above 200 bp were observed using the N5N6 CCExo system.

Based on substantial increase in genome modification in CCExo enhanced CRISPR system we aimed to determine the potential off-target activities on a genome-wide scale by implementing high through-put CIRCLE-seq method^49,50^. HEK293 and K562 cells isolated genomic DNA was used from which genomic libraries were prepared that were then subjected to VEGF gRNA and TRBC1 gRNA mediated cleavage (Supplementary Figs. 16–18). By Illumina NGS obtained CIRCLE-seq data analysis we discovered that only in VEGF site in K562 cells the number of hits for predicted off-target sites was elevated in Cas9+ExoIII or CCExo treated libraries pointing to higher off-target activity, but the number of counts for the highest predicted site was the same in Cas9 as in CCExo digested sample (Supplementary Fig. 17). In other samples no difference (hits and counts) was observed between Cas9 or CCExo treated samples (Supplementary Figs. 16,18). While lower count numbers were detected in some samples, they are nonetheless in accordance to published literature^49,51,52^. CIRCLE-seq predicted off-target sites were PCR amplified and subjected to NGS analysis, wherein no difference in off-target CRIPSR activity was seen in CCExo compared to Cas9 treated cells, even in some K562 cell samples, where higher off-target activity was predicted by CIRCLE-seq analysis (Fig. 3e).

Next, to test the applicability of a coiled-coil-recruited exonuclease gene editing enhancement to other CRISPR systems, a coiled-coil tethered version of a type V CRISPR^18^ system was produced. *Lb*Cas12a was fused to the N5 peptide enabling recruitment of N6-ExoIII. Based on T7E1 assay we confirmed that the CCExo augmented gene inactivation also in this type V CRISPR system (Supplementary Fig. 19).

### CCExo for cancer gene disruption

**Fig. 4:**
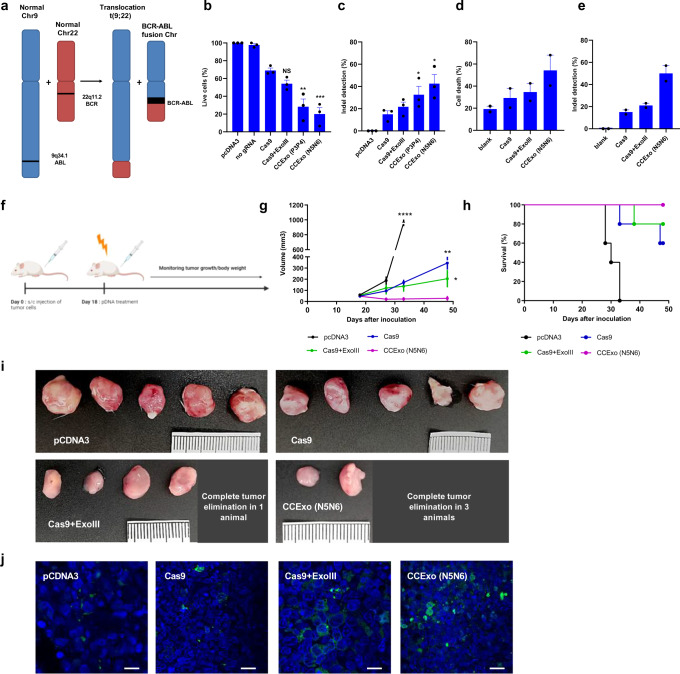
*BCR-ABL1* editing via CCExo. Schematic representation of *BCR-ABL1* fusion chromosome development (**a**). K562-fLUC cell death following pDNA electroporation. Cell viability was determined via bioluminescence signal intensity, wherein the value of total flux (p/s) bioluminescence of pcDNA3 electroporated cells was set to 100%. Cell death was determined 72 h post electroporation (**b**). Indel mutation quantification measured via the T7E1 assay in K562 cells edited with CCExo targeting the *BCR-ABL1* region 72 h post electroporation (**c**). Data represent three independent experiments (*n* = 3). *P < 0.05, **< 0.01, ***< 0.001 and ****P < 0.0001. All P values are from ordinary one-way ANOVA followed by Tukey’s multiple comparisons test compared to Cas9 values. Data are presented as mean values ± SEM as appropriate (**b**–**c**). PBMCs of CML patients showed enhanced cell death following CCExo RNP electroporation. Cell death was determined by TUNEL assay, where cell death was normalized to an apoptosis induced control cell sample. Dots represent values corresponding to individual patient donor PBMCs (*n* = cells from two CML patients) (**d**). Indel mutation quantification measured via the T7E1 assay in PBMCs of CML patients edited with CCExo targeting the *BCR-ABL1* region 72 h after the electroporation. Data represent two independent patient donor PBMCs (*n* = 2). **P < 0.01. All P values are from ordinary one-way ANOVA followed by Tukey’s multiple comparisons test compared to Cas9 values. Data are presented as mean values ± SEM as appropriate (**e**). Diagram showing the timeline of in vivo experiments using SCID mice with xenograft K562 cancer model for potential anti-tumor therapeutic application determination. At day 18, pDNA (50 μg/animal) was intratumorally electroporated. At day 50, the experiment was terminated (**f**). Cumulative representation of tumor volumes, treated with indicated plasmids. Data are presented as mean values ± SEM as appropriate. All *P* values are from unpaired *t* test with Welch’s correction, (*n* = 5 animals). *P < 0.05, **< 0.01 and ****P < 0.0001 compared to CCExo treated animals (**g**). Survival plot of animals involved in the experiment (**h**). Tumor size of K562 xenograft tumors, taken from the mice at the end of the experiments (**i**). TUNEL assay determining apoptotic cell death (green-Brdu-Red positive cells, blue-DAPI stain) in tumors tissue slides, derived from animals, treated with CCExo. A representative image from individual treated animal is provided. Scale bar, 50 μm (**j**).

As the CRISPR/Cas system is gaining use as a therapeutic option^53,54^ the augmented efficiency of CCExo might be used as a welcome addition for specific therapeutic applications as the specific gene target in cancer cells could be determined for each patient. We decided to apply CCexo system to target chronic myelogenous leukemia (CML). CML is a rare hematopoietic stem cell disease wherein cells harbor the characteristic “Philadelphia chromosome” (Ph), resulting from a translocation between the *BCR* and *ABL1* genes (Fig. 4a), forming the p210 or p190 isoforms of BCR-ABL1 tyrosine kinase which drive cancer cell proliferation^55^. The fusion gene *BCR/ABL1* is present in 95% of CML patients and ~30% in ALL (acute lymphoblastic leukemia) and has been already precisely targeted and disrupted using the CRISPR/Cas system^56^. To assess whether the K562 cell line is a suitable cell model for CML, we PCR amplified and sequenced the predicted *BCR-ABL1* break junction and designed gRNA precisely targeting this cancer-specific junction (Supplementary Fig. 20a). By exposing K562 cells to therapeutic tyrosine inhibitors^57^ we established that cell death is dose dependent and therefore K562 represents a good model for CML therapy with BCR-ABL as a survival factor (Supplementary Fig. 21). We proceeded with CCExo plasmid electroporation of K562-fLUC-eGFP expressing cells. *BCR/ABL1* fusion gene was successfully disrupted, which was observed through augmented cell death, determined with bioluminescence measurement (Fig. 4b), LDH release (Supplementary Fig. 20b) and TUNEL assay (Supplementary Fig. 20c) that showed ~ 80% cell death for N5-N6 CCExo. Again, the superiority of CCExo was observed compared to the regular CRISPR/Cas system and confirmed also via T7E1 assay (Fig. 4c).

Further, to test specificity of the *BCR/ABL1* gene targeting via CCExo system, we electroporated a co-culture of K562 and Jurkat, a T cell line lacking the specific translocation, where we found that Jurkat cells were not affected and that treatment was highly specific for K562 cells harboring the *BCR/ABL1* gene (Supplementary Fig. 20d–e). Finally, T7E1 of two predicted off-target sites for *BCR-ABL1* gRNA showed that DNA deletions occurred only at the targeted genomic site (Supplementary Fig. 20f), whereas on-target activity was determined (Fig. 4c).

Based on these results, we developed a K562 xenograft cancer model in SCID mice that were electroporated with appropriate plasmids (Fig. 4e). We observed that all CRISPR/Cas treatment modalities targeting the *BCR/ABL1* fusion gene were beneficial with respect to the tumor volume, as assessed 30 days post tumor electroporation (Fig. 4f, Supplementary Fig. 22). While all the tumors remained in animals treated with the standard CRISPR system comparable to the control group, albeit with a reduced tumor size, one tumor in the group treated with Cas9+ExoIII was completely eradicated (Fig. 4h; Supplementary Fig. 20c). Treatment with CCExo, tethered by a strong coiled-coil peptide pair resulted in a strongly diminished tumor volume with three out of five tumors being completely eradicated (Fig. 4h; Supplementary Fig. 20d). TUNEL assay of cancer tissue slides revealed that apoptosis of cancer cells was most abundant in CCExo treated animals (Fig. 4i), confirming the potential therapeutic benefit of enhanced CC-based CRISPR/Cas system, which ensured prolonged survival of experimental animals (Fig. 4g).

Since primary human blood cells are difficult to transfect with plasmid DNA, RNPs composed of isolated recombinant proteins (Supplementary Fig. 6) combined with synthetic gRNA were used. RNP-mediated targeting of the *BCR/ABL1* genomic region was first tested in the K562 cell line, where we confirmed the functionality of recombinant proteins (Supplementary Fig. 20g). CML patient-derived PBMCs electroporated with RNP complexes demonstrated best performance of CCExo in comparison to other tested variants (Fig. 4d, Supplementary Fig. 23). As the CRISPR/Cas system has been recently also used in CAR-T cell^58^ generation, we aimed to test implementation of CCExo for the support of cancer immunotherapy to inactivate a checkpoint receptor in therapeutic cells. CCExo RNPs, comprising gRNA targeting PD-1 gene^59^ were delivered into T cells in order to achieve PD-1 knock out. By FACS analysis and T7E1 indel detection assay of treated T cells we found that the fraction of PD-1 negative cells substantially increased when PD-1 gene disruption was targeted by CCExo RNPs (Supplementary Fig. 24).

## Discussion

Due to the strong impact of the CRISPR/Cas system in diverse fields of biomedical applications, its paramount efficiency is highly desired. In this report, we designed an enhanced version of the CRISPR/Cas system, where Cas9 and ExoIII were coupled via a noncovalent CC-mediated tethering. This resulted in a substantially increased rate of gene inactivation, compared to the standard CRISPR/Cas system or co-expression of Cas9 with exonucleases (Supplementary Fig. 25). Coiled-coils which are often used in synthetic biology as building blocks^32^ have been used in dCas9-mediated transcription^36^ and, in this report, as a tool to improve gene editing efficiency. CCExo demonstrated augmented, depending on the target, up to 7-fold higher efficiency of gene inactivation, compared to the Cas9 protein alone or co-expression of Cas9 with an exonuclease. While covalent fusion of Cas9 has been effective for some enzymes, such as FokI or reverse transcriptase, fusion of Cas9 with ExoIII did not result in a substantial increase. However, the efficiency of gene disruption was substantially improved by recruiting ExoIII to Cas9 via genetic fusion of each of the partners to a complementary CC dimer-forming peptide. This improvement concerns both the rate of indel mutation, as well as the length of deletions, which spanned up to 200 nucleotides for N5N6 CCExo. DNA recession provided by exonuclease prevented re-ligation of the exposed DNA ends, which would otherwise represent an idle cleavage-religation cycle. The proximity of ExoIII to the cleavage site facilitates in situ processing, leading to higher efficiency of indel formation. CC-mediated tethering increases the local concentration of exonuclease at the target site and also likely enables faster dissociation of Cas9 based on the observed higher cleavage efficiency. Local presence of the exonuclease allows more extensive processing of DNA at the target site, as demonstrated by the increased deletion size, which is more likely to result in inactivation of the target gene. The CCExo-based editing tool can apparently be widely applicable to different cell types and DNA targets. Editing was demonstrated also in somatic adult cells within a living animal, again with substantially higher efficiency compared to the standard CRISPR/Cas^60^. Increased gene inactivation was also observed for Cas12a, demonstrating the applicability of CCExo for different CRISPR systems. Finally, CCExo implemented as a plasmid or RNP was assessed for potential therapeutic application on the *BCR/ABL1* fusion gene in CML patient-derived cells. The absence of additional off-target effects can be explained by the requirement for Cas9-mediated cleavage that is not modified by coupling to the exonuclease and could be further increased by Cas9 variants with the increased fidelity.

Animal cancer model and apoptotic cell death detection in CML cells showed that CCExo surpassed the anti-cancer function of standard Cas9 or co-expression of Cas9 and ExoIII. The CCExo approach for treating CML could represent an alternative to tyrosine kinase inhibitors, since the latter may become inefficient due to susceptibility to point mutations^61^. On the other hand, any mutations that might affect the specificity of gRNA targeting *BRC/ABL1* fusion gene could be promptly implemented into the new design of targeting gRNA that would allow efficient binding and cleavage of CCExo, regardless to kinase inhibitor resistance due to point mutations in the targeted genomic region. CCExo-mediated cancer treatment could be extrapolated not only to the *BCR-ABL1* fusion gene but likely to many others. According to the COSMIC database (cancer.sanger.ac.uk) there are >300 reported cancer-specific fusion genes, responsible for cancer development in somatic cells. Besides cancer genes, ClinVar database (ncbi.nlm.nih.gov/clinvar) lists more than 72,000 pathogenic variants with clinical significance that drive genetic diseases, where ~14,000 are noted as likely pathogenic and ~38,000 as pathogenic. CCExo approach could be used to target genes, where duplications (~5500), insertions (~7700) and indel (~1200) mutations are the cause of the diseases.

Our results show that the CCExo system may be applied using a single or dual plasmid strategy. While single plasmid expression is beneficial in terms of optimal delivery, dual plasmid strategies have been widely applied for genome editing, such as split Cas systems^62,63^, recruitment of diverse activator domains^64^ and base and prime edit systems fused to intein domains^65,66^. Dual plasmid delivery bears some concerns as several components need to be carefully adiminstered but on the other hand allows several advantages such as packaging of large multi-domain components into size limited viral vectors, titration of system components and system modularity allowing mixing and matching of diverse or additional protein domains with which we can tailor the system to achieve diverse downstream effects. On the other hand an adenoviral delivery system, capable of delivering all components of the CCExo system in one vector^48^ can be applied for potential therapeutic options.

RNP implementation of CCExo could be used for other relevant biomedical applications, such as e.g. in cancer immunotherapy. Therapeutic CAR-T cells^67,68^ that lack endogenous complexes to recognize foreign cells could redirect treatment to allogenic transplant. Further, infusion of CRISPR-modified PD-1 KO T cells into cancer patients could overcome the checkpoint inhibitory effects of tumors on the immune system. Several clinical trials, using CRISPR to treat cancer have concluded as safe and feasible^69^. Our results suggest that CCExo enhanced genome engineering can be used in biotechnological as well as in translational medical applications for targeting the diseased gene for morbidities with genetic etiology, however additional research is needed to fully establish CRISPR/Cas system as a safe therapeutic choice.

## Methods

### Cell cultures

The human embryonic kidney (HEK) 293 and mouse Neuro-2A cells were purchased form American Type Culture Collection (ATCC). The 293/GFP cell line, which stably expresses GFP, was purchased from Cell Biolabs. Cells were cultured in DMEM (Invitrogen Life Technologies) supplemented with 10% (v/v) heat-inactivated FBS (Invitrogen Life Technologies). Jurkat cells and K562-fLUC cells (kind gift from Sebastien Wälchli) were grown in RPMI160 medium with 10% FBS. Cells were cultured at 37 °C in 5% CO2. For cell transfection, jetPEI (Polyplus transfection) was used according to their protocol.

Samples from newly diagnosed CML patients were obtained after translocation t(9;22)(q34;q22) and a fusion gene *BCR/ABL1* transcript was confirmed. Mononuclear cells from CML patients were isolated via Ficoll® Paque gradient centrifugation. Samples were obtained with informed consent, and according to the study protocol approved by National Medical Ethics Committee of the Ministry of Health, Republic of Slovenia (permit number 0120-600/2019/3).

T cells for PD-1 gene disruption were obtained from healthy donors. Samples were obtained with informed consent, and according to the study protocol approved by the National Medical Ethics Committee (0120-21/2020/4). T cells were isolated from PBMCs by Ficoll® Paque gradient centrifugation. Afterwards Miltenyi PanT cell isolation kit was used. CD3 positive cells were maintained in RPMI medium, supplemented with 10% FBS, 25 μl/ml of ImmunoCult^TM^ Human CD3/CD28 T cell activator (Stemcell ^TM^) and 10 ng/ml of human IL2 (Preprotech) for five days before the electroporation. Four days after electroporation, cells were subjected to T7E1 assay and FACS analysis.

### Plasmids

Target sites for genome editing were determined by using the CRISPR Design Tool from MIT or Benchling. sgRNAs were cloned with BstXI/NheI restriction/ligation into the pgRNA-humanized plasmid (Addgene plasmid 42230). Cas9 sequence was obtained from pX330-U6-Chimeric_BB-Cbh-hSpCas9 vector (Addgene plasmid 46911) and cloned via Gibson assembly into pcDNA3.1 vector. The SV40 large T-antigen nuclear localization sequence (NLS) and sequences for coiled coils were introduced into the constructs with PCR. Sequence for human exonuclease I (ExoI; Genscript RC200547), human Werner syndrome ATP-dependent helicase (human WRN; Addgene 46038), mouse TREX2 (Addgene 40210), E.coli exonuclease III (ExoIII; Addgene 46884) and human flap structure-specific endonuclease 1 (human FEN1; Addgene 35027) were transferred into pcDNA3.1 plasmid via Gibson assembly. For recombinant protein isolation, pET-28b-Cas9-His (Addgene plasmid 47327) was used as a template, where again coiled-coils were introduced with PCR. For Cas12a the pTE4398 (Addgene; plasmid 74042) vector was used.

### Cell electroporation

K562-fLUC cells were electroporated using Gene Pulser Xcell^TM^ Electroporation System (Biorad). Briefly, 3×10^6^ cells in 300 μl RPMI1640 medium were placed into 0.4 cm cuvette. 25 μg of designated plasmid DNA (Cas9: ExoIII: gRNA plasmid DNA ratio = 1:1:1) was added to cell suspension and incubated for 15 min at room temperature. Afterwards electroporation was carried out, using the following settings: 875 V/cm, 500 μF, ∞ resistance. After that, the cells were centrifuged for 30 s at 13.400 RPM. The cell pellet was left undisturbed for 20 min. Afterwards, the cell pellet was transferred to fresh medium.

When using ribonucleoprotein complexes for genome editing, K562 cells or PBMCs from CML patients (1–2 × 10^7^ cells/ml) were electroporated by Neon electroporation system (ThermoFisher Scientific), using 10 μl electroporation tips (electroporation parameters for K562: 1450 V voltage, 10 ms pulse width, 3 pulses; for CML PBMCs: 1600V, 10 ms, 3 pulses). Cas9RNP formation (for CML patient cells 500 nM of Cas9 or exonuclease protein and 1000 nm of sgRNA; Synthego, for K562 cells: 100 nm of Cas or exonuclease protein and 900 nm for sgRNA; Synthego) was carried out for 20 min at room temperature in R buffer.

T cells for PD1 disruption (1–2 × 10^7^ cells/ml) were electroporated by Neon electroporation system (ThermoFisher Scientific), using 100 μl electroporation tips (electroporation parameters: 1100 V voltage, 10 ms pulse width, 2 pulses). Cas9RNP formation (300 nM of Cas9 or exonuclease protein and 1400 nm of sgRNA; Synthego) was carried out for 20 min at room temperature in R buffer. PD-1 negative cells were detected via FACS, using CD279 (PD1)-PE-Vio770, human antibodies from Miltenyi Biotec (130-117-698). T cells were stained with anti-human CD3 Antibody, eFluor405 from Miltenyi Biotec (130-113-128).

### Cytotoxicity determination

To determine cell death several methods were used. LDH cytotoxicity assay (ThermoFisher Scientific) and TUNEL assay (BrDU-Red; ab66110; Abcam) were carried out according to the manufacturer’s protocol.

Determination of bioluminescence (BLI) loss was used as a marker for cell death in K562-fLUC cells. After appropriate treatment, 500 μM D-luciferin (Xenogen) was added to cells. BLI was measured using IVIS® Lumina Series III (Perkin Elmer). Data was analyzed with Living Image® 4.5.2 (Perkin Elmer). From Average Radiance values (ARV) the percentage of specific lysis was calculated using formula: % specific lysis = 100*(spontaneous death ARV-test ARV)/(spontaneous death ARV-maximal killing ARV).

### Cas9 and Cas9 variants protein isolation

*E. coli* NiCo21(DE3) or Rosetta competent cells (New England Biolabs) were transformed with p-ET28b- Cas9-NLS-6xHis (Addgene 47327) or constructs encoding Cas9-N5, ExoIII or ExoIII-P3 variants and grown at 37 °C overnight (160 rpm) in LB medium containing kanamycin (50 µg/mL). Bacterial cultures were transferred to LB medium at OD of 0.1, grown at 37 °C until OD reached 0.6 and induced with 0.25 mM IPTG. After induction, cells were cultured for 7 additional hours at 30 °C or 16 °C overnight in the case of Cas9-N5. The harvested cells were resuspended and lysed on ice with a lysis buffer. Cell lysis was completed by ultrasonication. Subsequently, cellular lysates were centrifuged at 16000 g (4 °C) for 30 min and soluble fractions were filtered through 0.2 µm filter units (Sartorius) and incubated overnight at 4 °C with Ni-NTA resin (Golden Biotechnology) previously equilibrated with buffer A (50 mM Tris buffer at pH 8.0, 150 mM NaCl, 10 mM Imidazole). After washing with buffer A and B (50 mM Tris buffer at pH 8.0, 150 mM NaCl, 10 mM imidazole and 50 mM Tris buffer at pH 8.0, 150 mM NaCl, 20 mM imidazole respectively) the bound fraction was eluted with buffer C (50 mM Tris buffer at pH 8.0, 150 mM NaCl, 250 mM imidazole). EDTA (1 mM) and Glycerol (10% v/v) were added to the eluted fractions. The samples were characterized by SDS-PAGE and western blotting.

### T7E1 assay

Genomic DNA from modified cells was isolated according to the manufacturer’s protocol of DNeasy Blood & Tissue Kit (Qiagen). 200 ng of isolated amplified genomic DNA (20 μl of purified PCR product in 1x NEB 2. Buffer (NEB)) was then denatured and reannealed to form heteroduplexes by using the next program on Veriti Thermal Cycler (life Technologies): 95 °C for 10 min; 95 °C to 85 °C ramping at –2 °C/s; 85 °C to 25 °C at – 0.25 °C/s; and 25 °C hold for 1 min. After reannealing process, products were treated with 1 μl of T7 Endonuclease I (NEB) and were analyzed on 10–15 % native PAGE gel. Gels were stained with SYBR Gold DNA stain (Life Technologies) for 30 min and were imaged with a DNA bioimaging system (Bio-Rad). Quantification was based on relative band intensities. Indel percentage was determined: 100 x (1 – (1 – (b + c)/(a + b + c))^1/2^), where a is the integrated intensity (determined by using ImageJ 1.53k software) of the undigested PCR product, wherein b and c are integrated intensities of cleaved PCR product.

Some PCR amplicons were also send for Sanger sequencing and then subjected to TIDE analysis^70^ or ICE CRISPR Analysis Tool from Synthego to determine the degree of genome editing.

### CIRCLE-seq analysis

To identify possible genome-wide off-target activities a CIRCLE-seq was performed step by step according to published literature^50^. Genomic DNA was isolated from HEK293 (3×10^7^) cells using the Monarch® Genomic DNA Purification Kit (NEB T3010) and eluted in water. 25ug of each DNA sample was sonicated on a Sonics Vibra-Cell VCX-600 Ultrasonic Processor using a microtip submersed in 200ul sample volume at 20% amplitude, 1 min 30 s (1 s on 2 s off) to obtain uniform DNA fragmentation resulting in 250 bp size fragments. DNA fragmentation was analyzed via agarose electrophoresis. All DNA cleanup was performed using Agencourt AMPure XP beads (Beckman Coulter) according to manufacturer’s protocol. To check for RNP cleavage efficiency, PCR amplicon, surrounding *VEGFα* gRNA (GGTGAGTGAGTGTGTGCGTG TGG) target site was in vitro cleaved using in vitro transcribed gRNA (90 nM), Cas9 buffer and recombinant Cas9 or Cas9-N5 (90 nM) and EXOIII or N6-EXOIII (90 nM). gRNA was in vitro transcribed using HiScribe^TM^ T7 ARCA mRNA Kit (NEB) with subsequent purification using Monarch^®^ RNA Cleanup Kit (NEB). Next, CIRCLE-seq library was prepared using Kapa HTP Library Preparation- PCR free Kit (Kapa Biosystems). Using HTP Library Preparation Kit components end repair with A-tailing was carried out. Next, stem loop adapter ligation using hairpin adapter (/5Phos/CGGTGGACCGATGATC/ideoxyU/ATCGGTCCACCGaT; a-phosphorothioate linkage) was ligated into A-tailed sheared DNA. To eliminate adapter-less DNA molecules Lambda exonuclease (5 U/μl; NEB) and Exonuclease I *E*.*coli* (20 U/μl; NEB) digestion was used. Next T4 PNK (10 U/μl; NEB) and USER (1 U/μl; NEB) enzymes were used to generate 4-bp overhangs. To achieve DNA circularization USER/PNK treated DNA was ligated using T4 DNA ligase (400 U/μl; NEB) and appropriate buffer. To degrade residual linear DNA, Plasmid-safe ATP-dependent DNAse (10 U/μl; Epicenter), 25 mM ATP and reaction buffer were used. Next, circularized purified DNA was in vitro cleaved using RNPs as stated above. After that, Next-generation sequencing library was prepared. NEBNext Adapter for Ilumina (15 μM; NEB) were ligated to A-tailed cleaved circularized DNA, using KAPA DNA ligase (Kapa Biosystems). Next USER enzyme (NEB) was added to adapter-ligated DNA. Afterwards PCR was carried out using 10 μM NEBNext i5 and i7 Primer (NEB). qPCR using fw (AATGATACGGCGACCACCGAG) and rw (CAAGCAGAAGACGGCATACGAGAT) primer was used to quantify CIRCLE-seq libraries. NGS analysis was performed on Ilumina MiSeq (2x150 bp; app.5×10^6^ reads/sample) by Genewizz with 5%Phi-X Spike-in. FASTQ results were analyzed via CIRCLE-seq software available at *git clone* https://github.com/tsailabSJ/circleseq.git with hg38 reference genome. Software that were used are Phyton v2.7, BWA 0.7.17 and SAMtools 1.19. Based on the results, obtained with CIRCLE-seq, predicted off-targets sites were additionally subjected to further NGS analysis. 150bp long amplicons surrounding possible CRISPR/Cas9 cut site were sent to Genewizz for NGS using Ilumina sequencing platform. Identified matches obtained on CIRCLE-seq analysis are provided in Source Data File. Python v 3.7 and Matplotlib 3.5 software were used for CIRCLE-seq Manhattan plot visualization.

### Next generation sequencing analysis

To perform deep sequencing 200 or 400 base pairs amplicons were generated via PCR, where genomic region of interest was amplified. The site of interest (CRISPR cut site) was placed within the first 100 bp. The samples were analyzed in MGH CCIB DNA CORE (200 bp amplicons for *MYD88* gene) or Genewizz (400 bp amplicons for *CD47*, *VEGFα* and *EMX1* gene), where they performed NGS with approximately 200.000 reads. The obtained data was analyzed using CRISPResso or Cas-analyzer^71,72^.

### In vitro cleavage assay

For the determination of cleavage capacity in vitro pcDNA3.1 vector, containing *TRAC1* gRNA was prepared. DNA targeting sequence (TGTGCTAGACATGAGGTCTA TGG) was introduced into plasmid backbone via Gibson assembly. For positive control (linearization of the plasmid) plasmid EcoRI-HF (NEB) restriction was used. For in vitro digestion, a 30 μl reaction was set up. First, NEB3.1 buffer, 30 nM sgRNA (Synthego), 30 nM Cas9 or its variants (Cas9-N5) ± 30 nM ExoIII or its variants (N6-EXOIII) and nuclease-free water were preincubated for 10 min at 25 °C to form RNPs. Then 3 nM of target DNA substrate; TRAC1-pcDNA3.1 was added and incubated at 37 °C for 15 min. Next, the mixture was heated at 65 °C for 10 min for RNP inactivation and then cooled down to room temperature. To evaluate subsequent ligation, 2 mM ATP and T4DNA ligase (NEB) with corresponding T4DNA ligase buffer, was added to the mixture and additionally incubated at 37 °C for 15 min. For DNA cleavage visualization agarose gel electrophoresis was used, immediately after the ligation incubation time.

### Immunoblotting

Proteins were separated by SDS-PAGE and transferred to a Hybond ECL nitrocellulose membrane (GE Healthcare). Blots were incubated with appropriate antibodies. Primary antibodies used for western blotting were as follows: mouse anti-CRISPR-Cas9 antibody (ab191468) from Abcam and rabbit anti-Alpha/Beta tubulin antibody (2148) that were purchased from Cell Signaling Technology. Secondary antibodies used for western blotting were goat polyclonal anti-rabbit IgG (ab6721) from Abcam respectively and goat anti-mouse IgG-HRP (sc-2005) from Santa Cruz. The immunoblots were visualized on G-box (Syngene).

### Mouse studies

All animal experiments were performed according to the directives of the EU 2010/63 and were approved by the Administration of the Republic of Slovenia for Food Safety, Veterinary and Plant Protection of the Ministry of Agriculture, Forestry and Foods, Republic of Slovenia (Permit Number U34401-3/2017/16, U34401-9/2020/9). Laboratory animals were housed in IVC cages (Techniplast), fed standard chow (Mucedola) and tap water was provided ad libitum. Mice were maintained in 12-12 h dark-light cycle at approximately 40–60% relative humidity with 22 °C of ambient temperature. All animals, used in the study, were healthy; accompanied with health certificate from the animal vendor.

To test the anti-tumor effect of modified CRISPR system, female 8–10 weeks SCID C.B-17/IcrHsd-*Prkdc*^*scid*^ mice (Envigo, Italy) were used for xenograft cancer studies.

5×10^6^ K562-fLUC cells were implanted into the right flank of the mouse. When tumors reached defined size (40 mm^3^), 50 μg of plasmid DNA, dissolved in 150 mM NaCl was injected and afterwards electroporated using Gene Pulser Xcell^TM^ Electroporation System (Biorad; settings: 900 V, 100μs, 6 pulses in 1 s interval). Body weight and tumor size via caliper measurement was performed every couple of days. Tumor burden was calculated using formula V = (a*b*c*Π)/6. Animals were humanely euthanized, when certain dimension of the observed tumor reached 12 mm, tumor ulcerated or when animals lost over 20% of its own body mass. For TUNEL assay, tumor samples were removed and fixated overnight in 10% neutral buffered formalin (Sigma Aldrich) and then embedded in paraffin (Leica Paraplast). The paraffin blocks were cut 7 μm thick with a rotation microtome RM 2245 (Leica). The tissue sections underwent deparaffinization and rehydration using xylene and different dilutions of ethanol (Sigma Aldrich). The tissues samples were then mounted on slides using Leica CV Mount (Leica) and subjected to TUNEL assay according to manufacturer’s protocol. DAPI was used for cell nuclei visualization. Visualization of tumor sections was carried out by confocal microscopy.

Mice (Hsd:ICR (CD-1); Envigo) were hydrodynamically injected into the tail vein with a volume of saline solution equivalent to 10% of body weight in 4–7 s, containing 60 μg of plasmid DNA using a 3-ml latex free syringe with 27 G needle (Beckton Dickinson) to test in vivo the applicability of modified CRISPR system. After one week, mice were sacrificed and liver samples were collected for further analysis (T7E1 assay). To determine Cas9 protein expression in hydrodynamically injected animals, additional injected animals were sacrificed three days later and liver was collected. Liver single cell suspension was prepared using gentleMACS^TM^ Dissociator (Miltenyi) and afterwards cells were stained with anti-Cas9 antibodies (ab191468, Abcam) and subjected to flow cytometry. For positive control B6(C)-*Gt(ROSA)26Sor*^em1.1(CAG-cas9*,-EGFP)Rsky^/J mice were used, with stabile expression of Cas9 protein. For Cas9 expression detection, mouse anti-CRISPR-Cas9 antibody (ab191468) from Abcam and Alexa Fluor® 488 goat anti-mouse IgG from Invitrogen (A11001) as a secondary antibody.were used.

To study CCExo protein toxicity, Balb/c mice were intravenously injected with 100 μg of Cas9 or Cas9-N5 protein with 100 μg of ExoIII or N6-ExoIII recombinant protein. One and eight weeks later blood was drawn and sera was used to determine IL6 (eBioscience, IL1β and TNFα cytokines. Ready-Set-Go ELISAs (eBioscience^TM^) were performed according to manufacturer’s instructions. Complete biochemical profile was determined using VetScan Mammalian Liver Profile reagent and Comprehensive Metabolic Panel rotor and analyzed on the biochemistry analyzer VetScan VS2 (Abaxis).

### Confocal imaging analysis

Images were acquired using a Leica TCS SP5 inverted laser-scanning microscope on a Leica DMI 6000 CS module (Leica Microsystems) equipped with a HCX Plane-Apochromat lambda blue 63× oil-immersion objective with NA 1.4. For excitation of eGFP and BrdU-Red, we have used a 50-mW, 488-nm diode laser; emission was detected between 490–550 nm for eGFP detection and between 570–600 nm for BrdU-Red detection. Images were analyzed with LAS AF Lite 1.0.

### Flow cytometry analysis

Flow cytometry analysis was performed with flow cytometer CyFlow (Partec) or the spectral flow cytometer Aurora with the blue, violet and red lasers (Cytek). Cells were washed with FACS buffer (PBS, 2% FBS) and resuspended in a 0.1 ml FACS buffer. For CD47 detection we used human CD47-FITC antibodies from Miltenyi Biotec (130-101-344). A 488 or 633 nm diode laser was used. Data were analyzed with FlowJo v10 software (Tree Star) and SpectroFlo (Cytek) software.

### Statistical analyses

Data are presented as means ± SEM. Exact *P* values are provided in the Source Data File. Some figures were created with Biorender.com (individual subscription) or Inkscape. Graphs were prepared using GraphPad Prism8.

### Reporting summary

Further information on research design is available in the Nature Research Reporting Summary linked to this article.

## Supplementary information

Supplementary Information

Reporting Summary

## Data Availability

Relevant Source data are provided with this paper in Source Data File and Supplementary Information file. CIRCLE-seq and all NGS data are deposited in NCBI SRA with accession number PRJNA849175. All other data are available from the authors of the paper upon request. Source data are provided with this paper.

## Source data

Source Data file

## Code availability

For CIRCLE-seq analysis we used previously published code^50^.
