# Supplementary material for: Coiled-coil heterodimer-based recruitment of an exonuclease to CRISPR/Cas for enhanced gene editing
Source: Nat Commun. 2022 Jun 23;13:3604. doi: 10.1038/s41467-022-31386-1 (PMC9226073; doi:10.1038/s41467-022-31386-1)
Supplement: Supplementary file 1 — Supplementary Information [file 41467_2022_31386_MOESM1_ESM.pdf]

Coiled-coil heterodimer-based recruitment of an exonuclease to CRISPR/Cas for enhanced gene editing

Supplementary information

Duško Lainšček[1, 2,#], Vida Forstnerič[1,#], Veronika Mikolič[3, 4], Špela Malenšek[1, 4], Peter Pečan[1,4], Mojca Benčina[1, 2], Matjaž Sever[3, 5], Helena Podgornik[3, 6], Roman Jerala[1, 2, *]

[1]Department of Synthetic Biology and Immunology, National Institute of Chemistry, Hajdrihova 19, Ljubljana, 1000, Slovenia.

[2]EN-FIST Centre of Excellence, Trg Osvobodilne fronte 13, Ljubljana, 1000, Slovenia.

[3]Department of hematology, Division of internal medicine, University Medical Centre Ljubljana, Zaloška 7, Ljubljana, 1000, Slovenia

[4]Graduate School of Biomedicine, University of Ljubljana, Ljubljana, 1000, Slovenia.

[5]Faculty of Medicine, University of Ljubljana, Korytkova 2, Ljubljana, 1000, Slovenia

[6]Faculty of Pharmacy, University of Ljubljana, Aškerčeva cesta 7, Ljubljana, 1000, Slovenia

# Authors contributed equally to this work

* Correspondence to: roman.jerala@ki.si; tel. no.: +38614760335; fax: +38614760300; National Institute of Chemistry, Hajdrihova 19, SI-1000 Ljubljana, Slovenia.

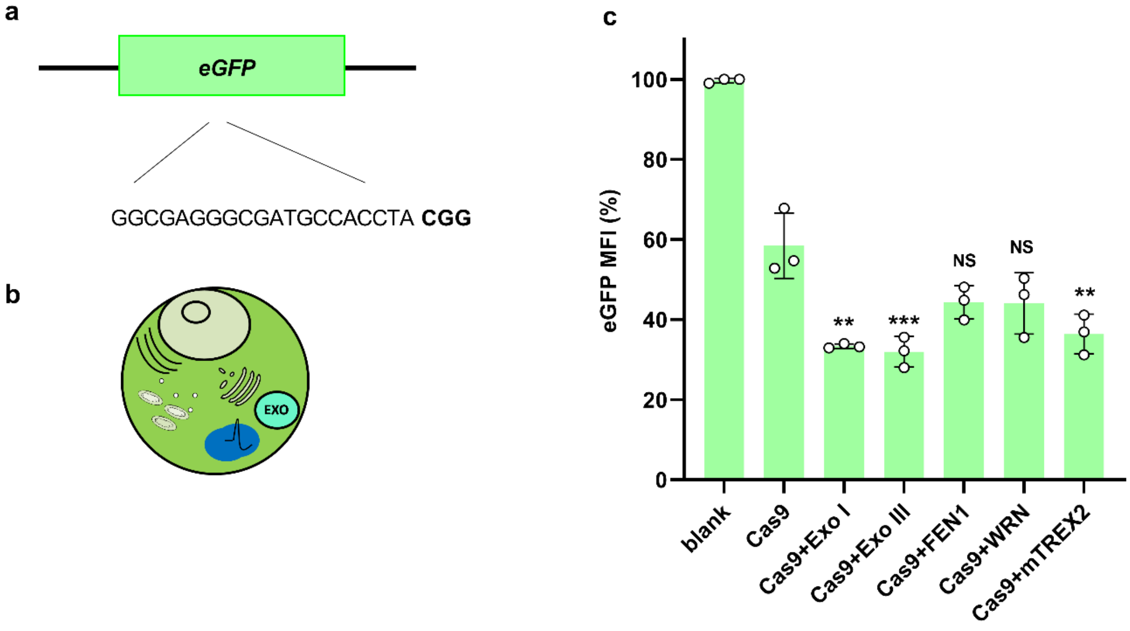

Supplementary Fig. 1: Coexpression of CRISPR/Cas9 and exonucleases in eGFP expressing cell line. DNA sequence of *eGFP* gene for designing gRNA for genome editing (a). HEK293-GFP stably expressing cells ($2*10^5$ cells/ml) were cotransfected with plasmids for Cas9, gRNA and certain exonucleases (600 ng of each component) (b). 48 hours after the transfection FACS analysis was performed to determine eGFP MFI to visualize *eGFP* gene disruption. MFI value of blank cells was denoted as 100% (c). Data present three individual separate experiments (n=3). *P< 0.05, ***P< 0.001. All P values are from ordinary one-way ANOVA followed by Tukey's multiple comparisons test compared to Cas9 values. Data are presented as mean values +/- SD as appropriate.

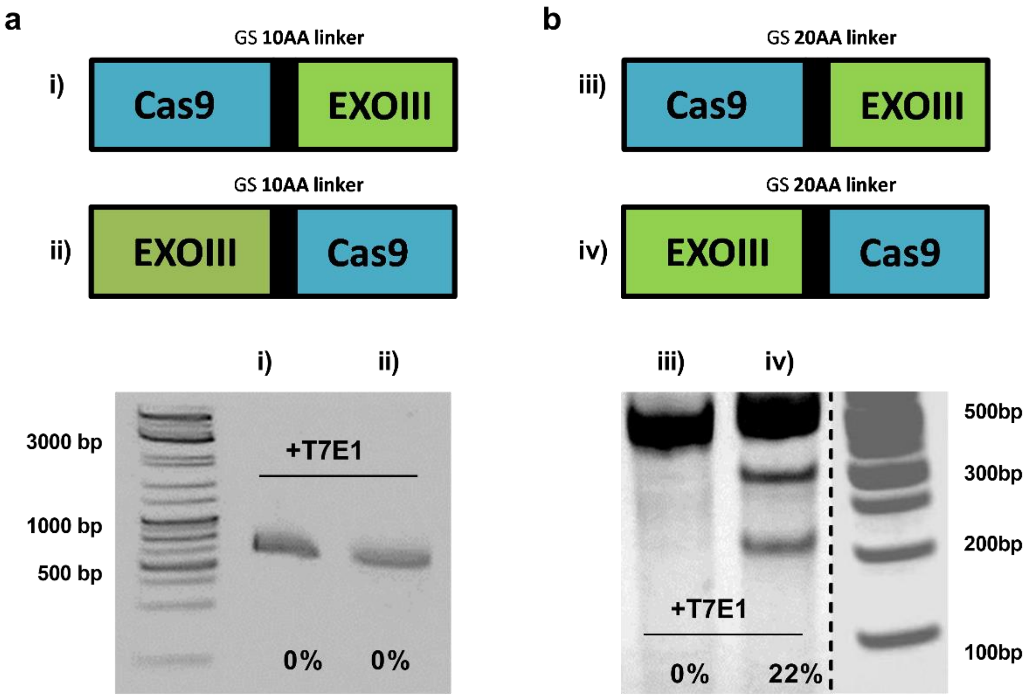

Supplementary Fig. 2: Constructs and an effect of *E.coli* ExoIII protein fusion to SpCas9. Fusion of *E.coli* ExoIII via 10 amino acid GS linker to N-terminus or C-terminus of the SpCas9 protein (upper panel). T7E1 assay on *MYD88* gRNA CRISPR/Cas system transfected HEK293 cells (bottom panel). HEK293 cells ($2*10^5$ cells/ml) were cotransfected with plasmids for gRNA and Cas9-ExoIII or ExoIII-Cas9, connected via 10 AA linker (600 ng of each component). 48 hours after the transfection, genomic DNA was isolated and T7E1 assay was performed. A representative image from three individual separate experiments is shown (a). Fusion of *E.coli* ExoIII via 20 amino acid GS linker to N-terminus or C-terminus of the SpCas9 protein (upper panel). T7E1 assay on *MYD88* gRNA CRISPR/Cas system transfected HEK293 cells (bottom panel). HEK293 cells ($2*10^5$ cells/ml) were cotransfected with plasmids for gRNA and Cas9-ExoIII or ExoIII-Cas9, connected via 20 AA linker (600 ng of each component). 48 hours after the transfection, genomic DNA was isolated and T7E1 assay was performed. A representative image from three individual separate experiments is shown. (b). Dashed line present merging of two agarose gel pictures.

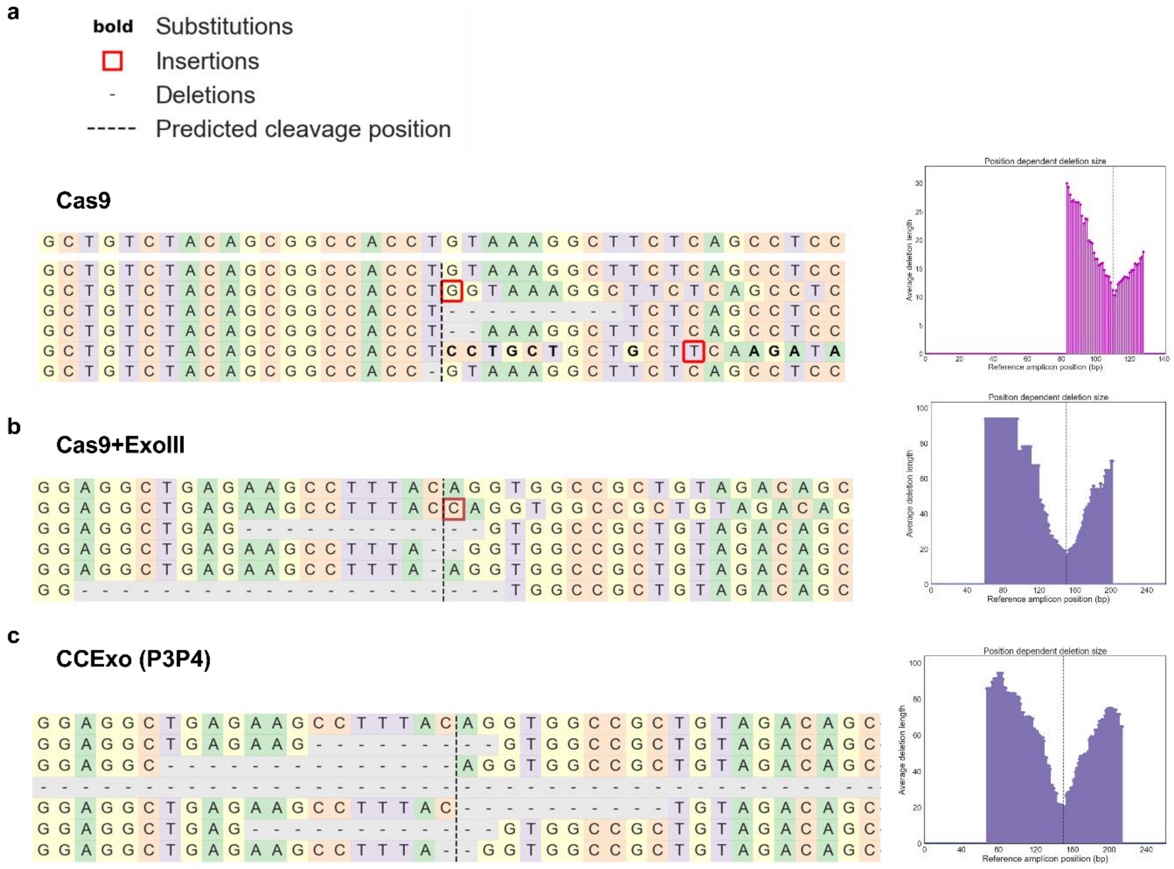

Supplementary Fig.3: Next generation sequencing of *MYD88* gRNA targeting PCR amplicon. HEK293 cells (2*10^5 cells/ml) were transfected with plasmids for gRNA, targeting *MYD88* gene and depicted CRISPR/Cas component. 48 hours after the transfection, genomic DNA was isolated and genomic region, surrounding gRNA targeting site was PCR amplified. 200bp PCR amplicon was subjected to NGS analysis. Data presents NGS results, where alleles surrounding predicted cleavage for Cas9 protein are shown with graphs, indicating position dependent deletion size (average deletion length in correspondence with amplicon position). Cells were transfected with gRNA and Cas9 only (a). Cells were transfected with gRNA, Cas9 and ExoIII (b). Cells were transfected with gRNA, Cas9-P3 and P4-ExoIII (c).

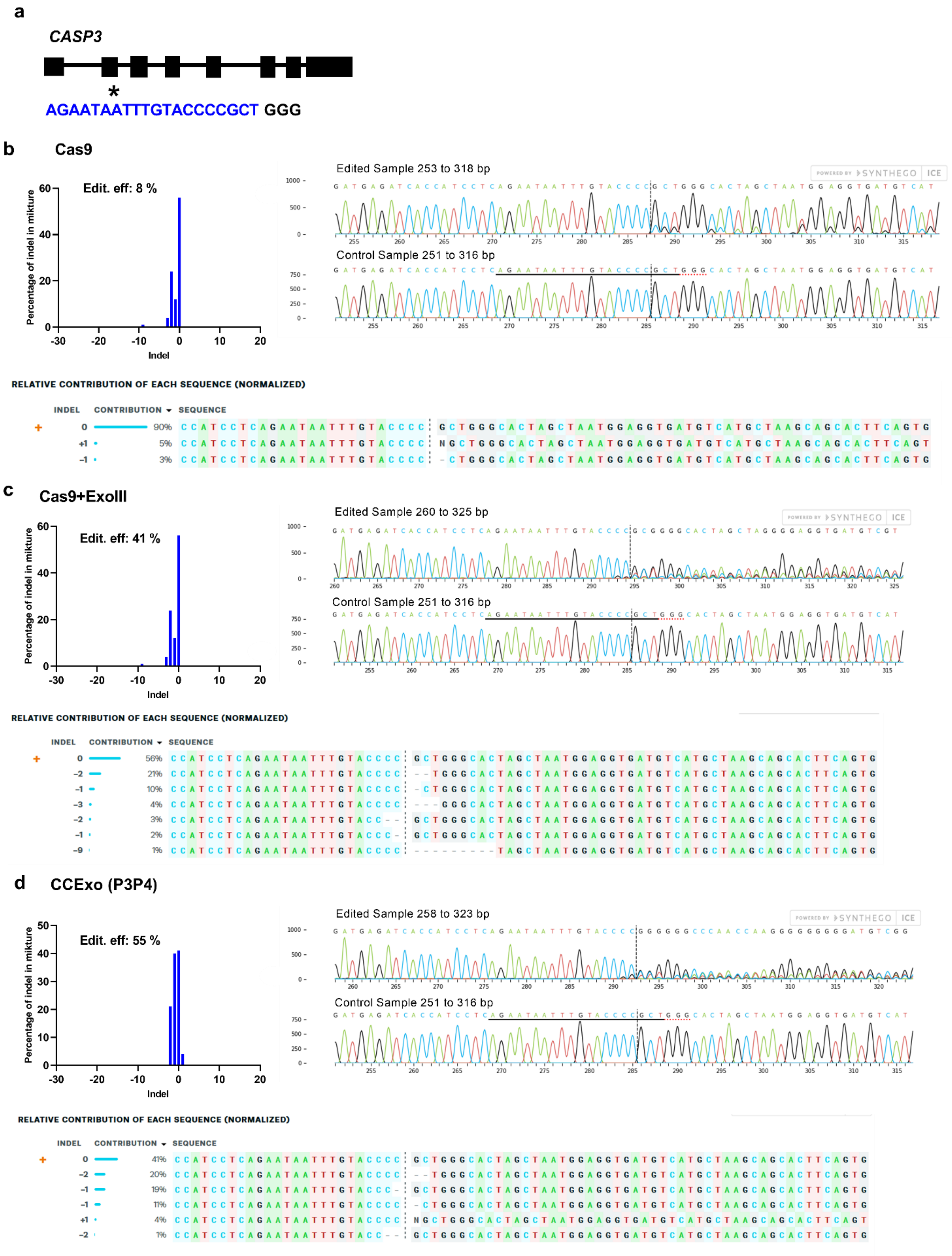

Supplementary Fig. 4: CCExo P3P4 enhances *CASP3* gene editing. DNA sequence of human *CASP3* gene for designing gRNA (blue-seeding sequence, black-PAM, * denote the

site of editing within the gene) for genome editing (a). HEK293 cells ($2*10^5$ cells/ml) were cotransfected with plasmids for Cas9, gRNA or CCExo (600 ng of each component). 48 hours later PCR amplicons (400bp) surrounding gRNA target site were obtained. Next, Sanger sequencing was carried out. Sequences were analyzed by ICE Synthego tool for genome editing. Analysis of regular CRISPR/Cas9 system action showing 8% editing efficiency (b). Analysis of coexpression of Cas9 and ExoIII CRISPR/Cas9 system action showing 41% editing efficiency (c). Analysis of CCExo (P3P4) action showing 55% editing efficiency (d).

Data present ICE Synthego tool analysis of Sanger sequences, where blue graph is showing editing efficiencies. Later, chromatogram of unedited and edited PCR amplicon is presented with subsequent allele distribution, surrounding predicted cleavage site, normalized to control (unedited PCR amplicon).

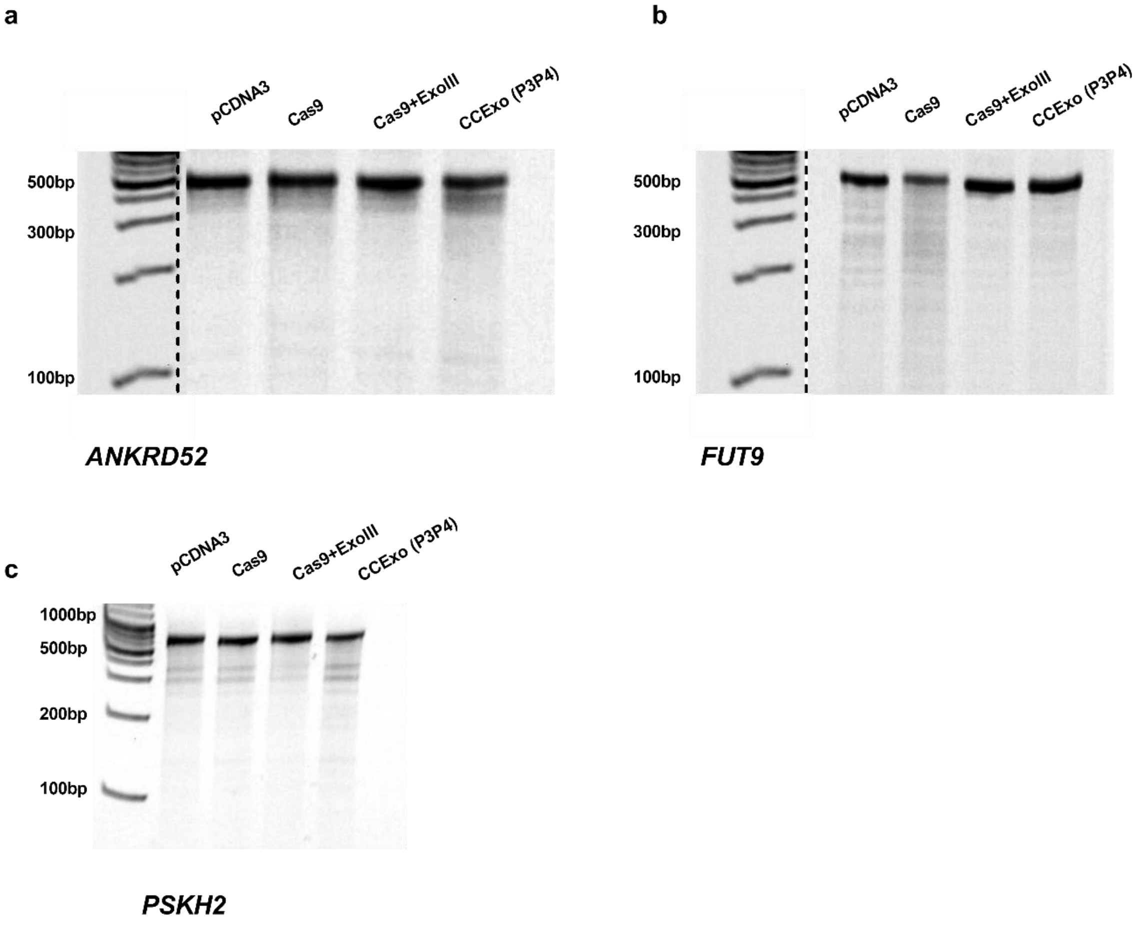

Supplementary Fig. 5: Analysis of cleavage at off-target sites, predicted for designed gRNA, targeting *MYD88* gene. HEK293 cells ($2*10^5$ cells/ml) were cotransfected with plasmids for Cas9, gRNA (targeting *MYD88* gene), ExoIII or CCExo (600 ng of each component). 48 hours later, genomic DNA was isolated, desired genomic region was PCR amplified and later T7E1 assay for indel detection of the top predicted off-target (*ANKRD52*, *FUT9* and *PSKH2* genes) sites was performed. Off-target sites were predicted using Benchling gRNA designer free software on-line tool. A representative image from two individual separate experiments is shown. Dashed line present merging of two agarose gel pictures.

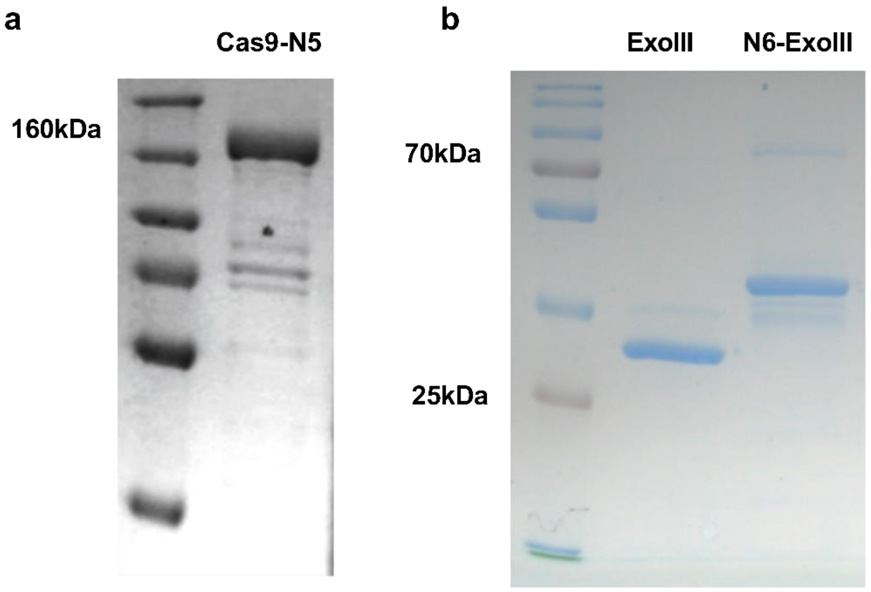

Supplementary Fig. 6: Production and purification of recombinant proteins for CCExo. SDS showing Cas9-N5 (a), ExoIII and N6-ExoIII isolation (b). A representative image from on of the isolation batch are shown.

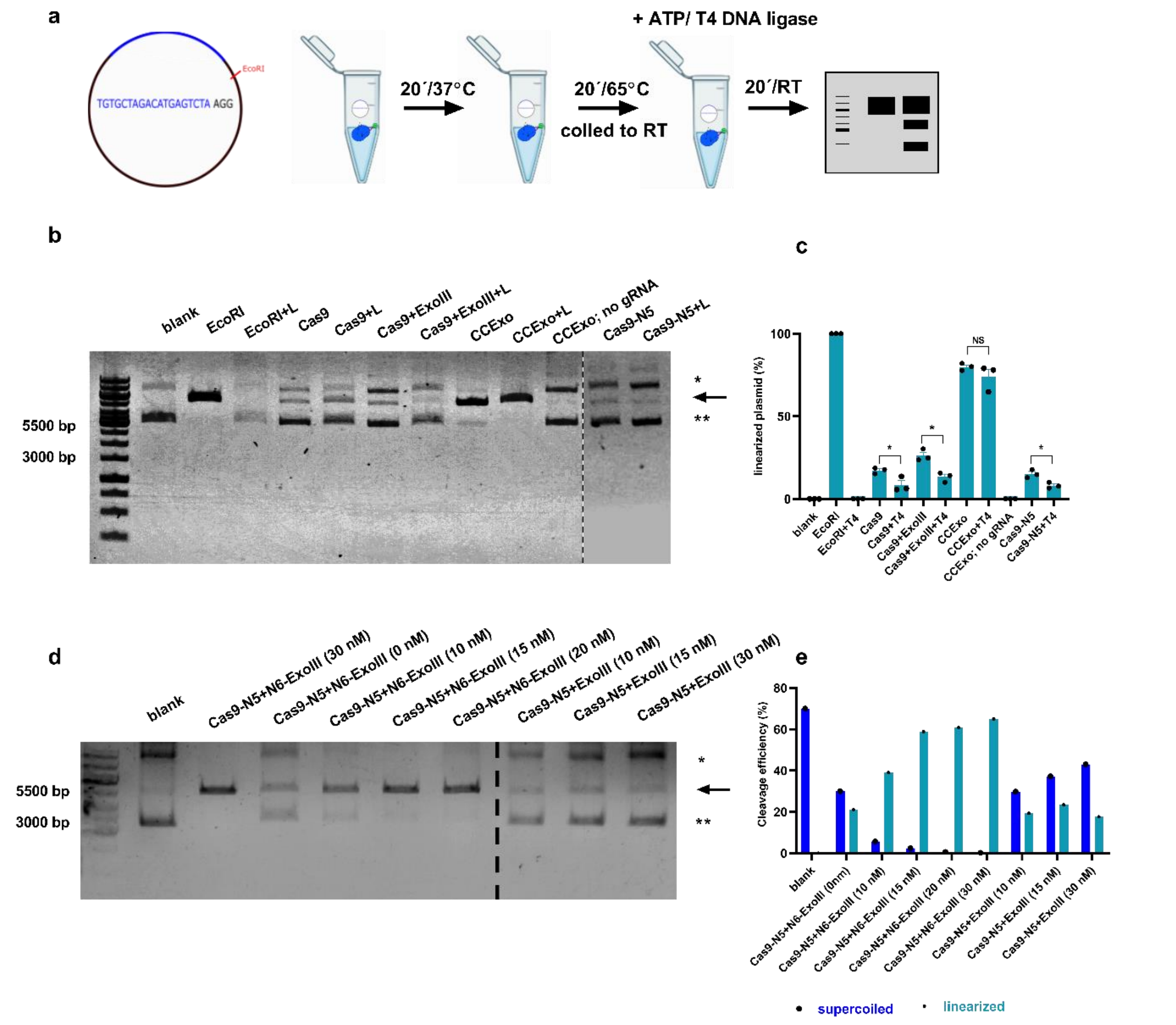

Supplementary Fig. 7: *In vitro* cleavage assay to analyze the efficiency of CCExo RNPs in DNA cleavage and prevention of cleaved DNA religation. Schematic presentation of the *in vitro* cleavage/religation assay. DNA template for *in vitro* cleavage was pcDNA3 plasmid with *hTRAC* gRNA targeting DNA sequence. Plasmid was treated with RNPs for 15 minutes, with subsequent heat inactivation before the addition of a T4 DNA ligase. To validate potential re-ligation of cut plasmid, T4 DNA ligase-based reaction was performed (+L). DNA cleavage was visualized using agarose gel electrophoresis (a). Agarose gel analysis of the TRAC-pcDNA3 plasmid linearization and ligation by EcoRI (positive control) or tested protein components. No re-ligation of the cut plasmid was observed in CCExo treatment (b). Quantification of the plasmid linearization/religation efficiency. EcoRI treated plasmid linearization was denoted as 100% (c). Linearization of the TRAC-pcDNA3 plasmid by Cas9-

N5 (30 nM) depending on the increasing amount of added N6-ExoIII or ExoIII protein analyzed by agarose gel electrophoresis (d). Quantification of the plasmid linearization efficiency compared to the remaining uncleaved supercoiled form of the plasmid due to the Cas9-N5 (30 nM) and N6-ExoIII (various concentration) action. Sum of band density of blank sample was denoted as 100% (e). Arrow, pointed at band, showing linearized plasmid, * corresponding to the relaxed circular form of a plasmid, whereas ** corresponds to the supercoiled (noncleaved) form of a plasmid. Dashed line present merging of two agarose gel pictures. Data are representative of three individual separate experiments (n=3). *$P < 0.05$. All P values are from ordinary one-way ANOVA followed by Tukey's multiple comparison test. Data are presented as mean values +/- SEM as appropriate.

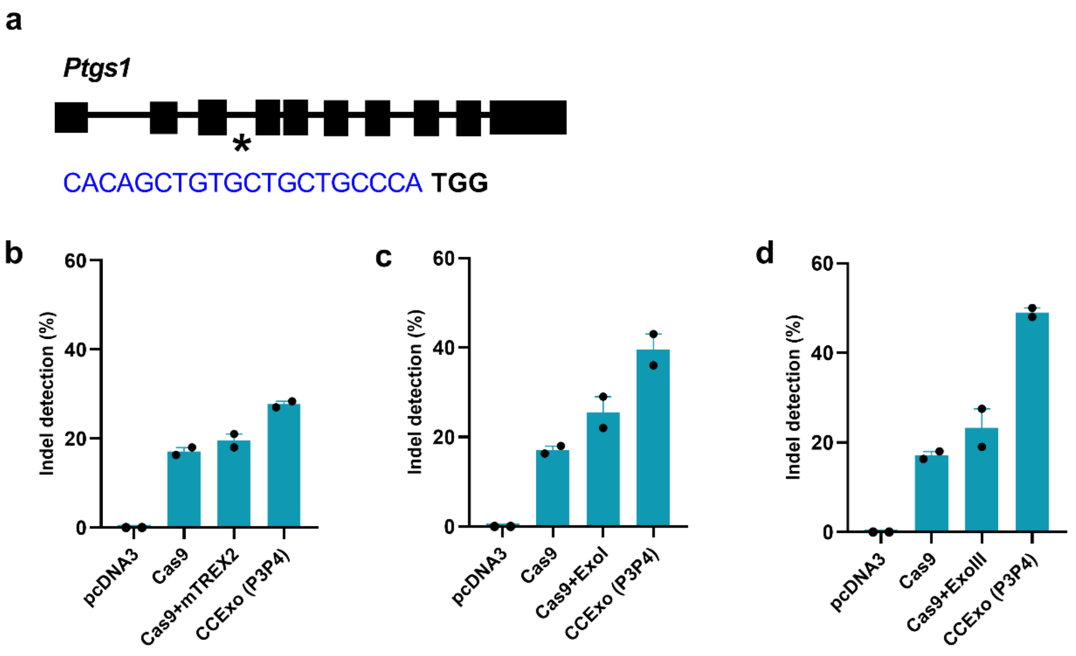

Supplementary Fig. 8: Increased indel formation by CCExo (P3P4) on mouse Neuro2A cell line. DNA sequence of mouse *Ptgs1* gene for designing gRNA (blue-seeding sequence, black-PAM, * denote the site of editing within the gene) for genome editing (a). Neuro2A cells (2*10^5 cells/ml) were cotransfected with plasmid for Cas9, gRNA or CCExo (600 ng of each component). 48 hours later, genomic DNA was isolated and appropriate genomic region was PCR amplified and later T7E1 assay for indel detection was performed. Indel detection quantification by T7E1 assay for editing with mouse TREX2 exonuclease (b) human ExoI (c) or *E.coli* ExoIII (d). Data present two individual separate experiments (n=2). Data are presented as mean values +/- SEM as appropriate.

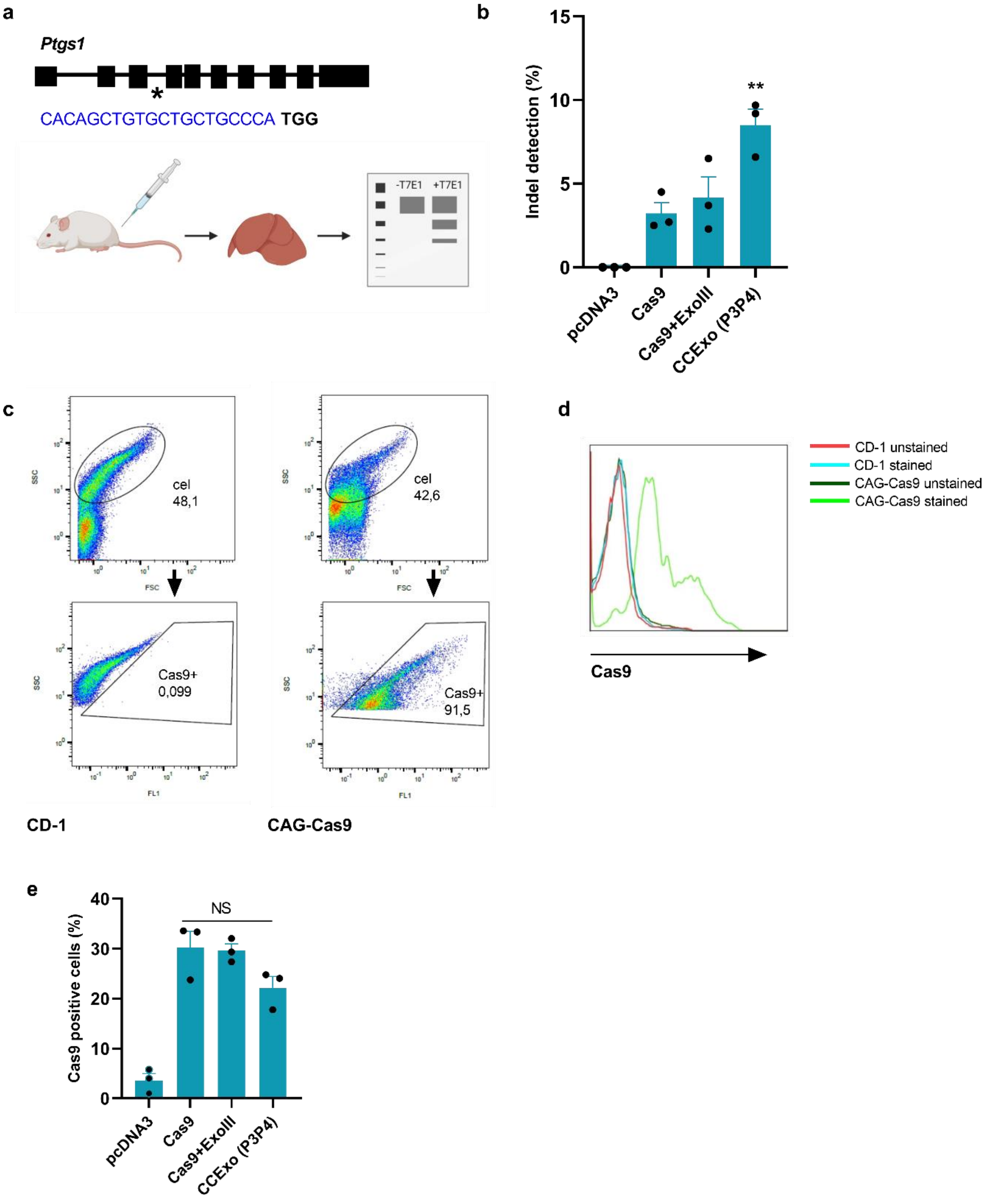

Supplementary Fig. 9: CCExo enhances gene editing *in vivo* in adult somatic cells. DNA sequence of mouse *Ptgs1* gene for designing gRNA (blue-seeding sequence, black-PAM, * denote the site of editing within the gene) for genome editing. Plasmids were hydrodynamically injected into mice. Seven days later, liver were harvested for isolation of genomic DNA. gRNA

targeting surrounding genomic region was PCR amplified and later T7E1 assay was carried out (a). Indel detection quantification by T7E1 assay for editing in mouse liver (b). FACS analysis and gating strategy for anti-Cas9 staining of liver cells of Cas9 negative (HSd-ICR) and Cas9 positive (CAG-cas9) mouse as a control to test Cas9 expression in hydrodynamically injected animals (c, d). Mice were hydrodynamically injected with depicted plasmids and 72 hours later, animals were sacrificed and liver cells were subjected to anti-Cas9 staining, determined via flow cytometry (e).

Data present three individual animals (n=3). **P< 0.01. All P values are from ordinary one-way ANOVA followed by Tukey's multiple comparisons test compared to Cas9 values. Data are presented as mean values +/- SEM as appropriate.

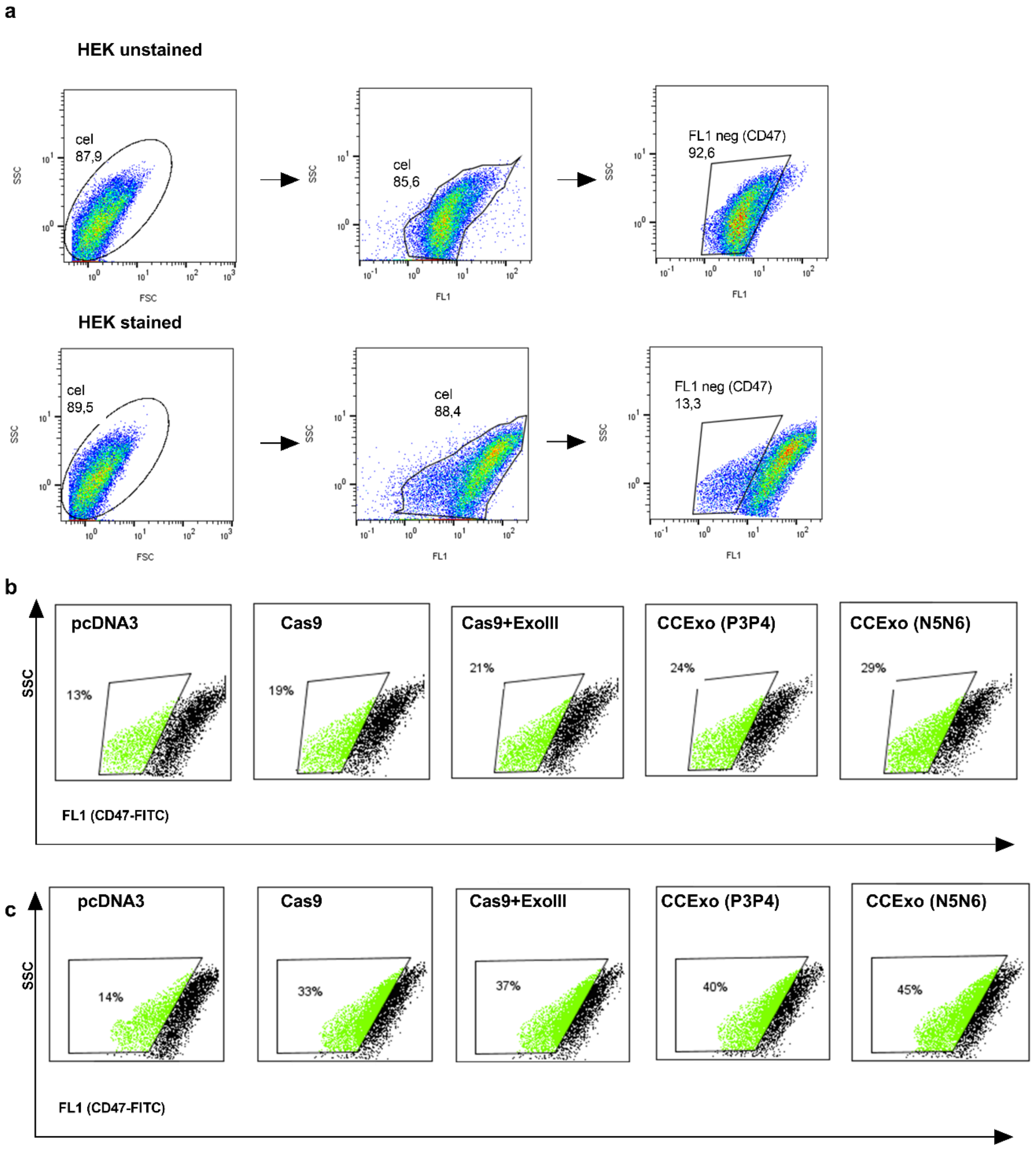

Supplementary Fig. 10: FACS analysis of CD47 expression in edited HEK293 cells. HEK293 cells (2*10^5 cells/ml) were cotransfected with plasmids for Cas9, gRNA (targeting *CD47* gene), ExoIII or CCExo (600 ng of each component). 48 hours later cells were stained against human CD47, determined via flow cytometry (a). FACS analysis showing increase in CD47 negative cells (green) when treated with CCExo plasmids, expressed together with gRNA1 (b) or when multiplexing (gRNA mix) was performed (c).

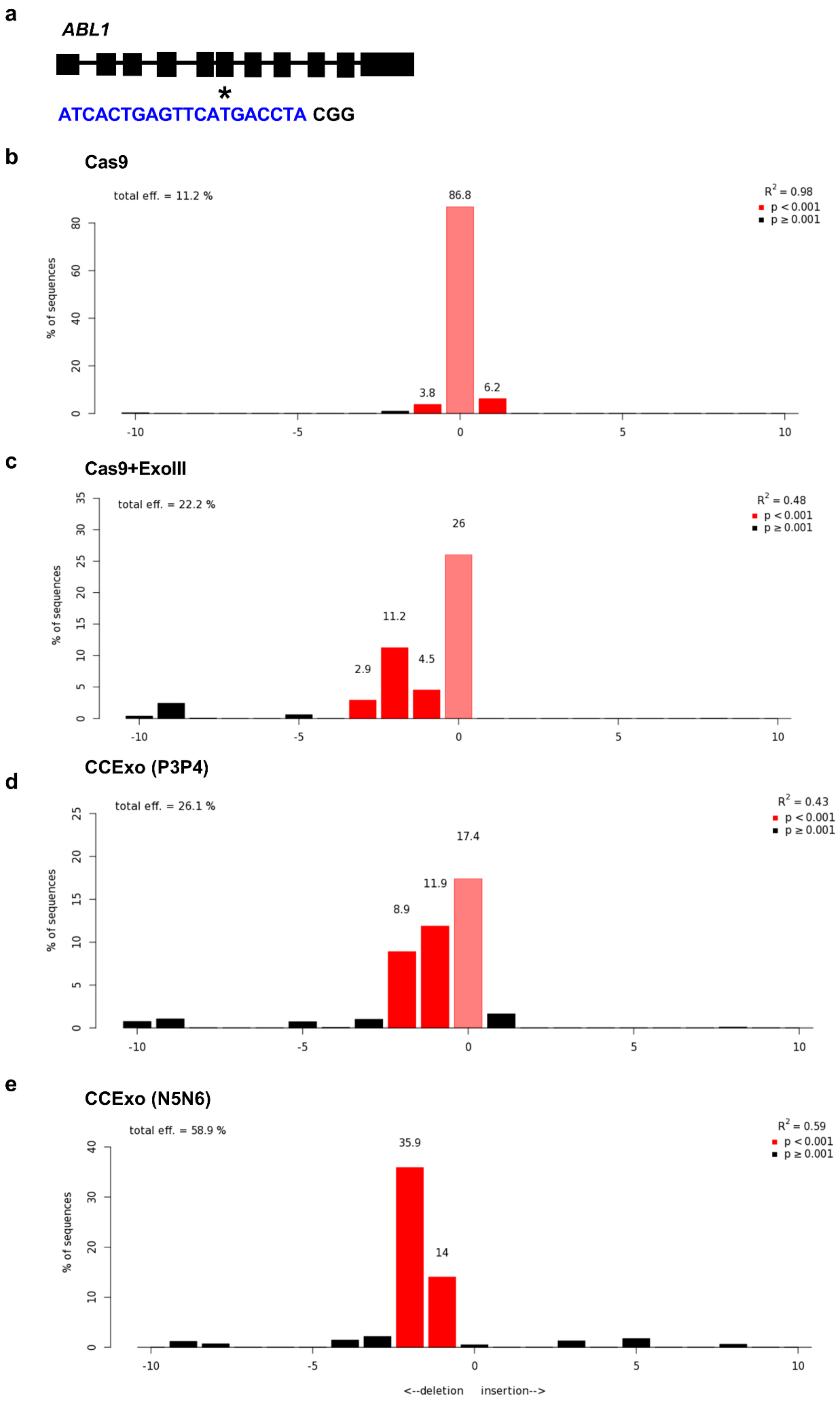

a
ABL1
*
ATCACTGAGTTCATGACCTA CGG
b
Cas9
total eff. = 11.2 %
86.8
3.8
6.2
R² = 0.98
p < 0.001
p ≥ 0.001
% of sequences
c
Cas9+ExoIII
total eff. = 22.2 %
26
11.2
2.9
4.5
R² = 0.48
p < 0.001
p ≥ 0.001
% of sequences
d
CCExo (P3P4)
total eff. = 26.1 %
17.4
11.9
8.9
R² = 0.43
p < 0.001
p ≥ 0.001
% of sequences
e
CCExo (N5N6)
total eff. = 58.9 %
35.9
14
R² = 0.59
p < 0.001
p ≥ 0.001
% of sequences
<--deletion insertion-->

Supplementary Fig. 11: CCExo enhances *ABL1* gene editing. DNA sequence of human *ABL1* gene for designing gRNA (blue-seeding sequence, black-PAM, * denote the site of editing within the gene) for genome editing (a). HEK293 cells (2*10^5 cells/ml) were cotransfected with plasmids coding for Cas9, gRNA or CCExo (600 ng of each component). 48 hours later PCR amplicons surrounding gRNA target site were obtained. Next, Sanger sequencing was carried out. Sequences were analyzed by TIDE analysis tool for genome editing. Analysis of regular CRISPR/Cas9 system action showing 11,2% editing efficiency (b). Analysis of coexpression of Cas9 and ExoIII CRISPR/Cas9 system action showing 22,2% editing efficiency (c). Analysis of CCExo (P3P4) action showing 26,1% editing efficiency (d). Analysis of CCExo (N5N6) action showing 58,9% editing efficiency (e).

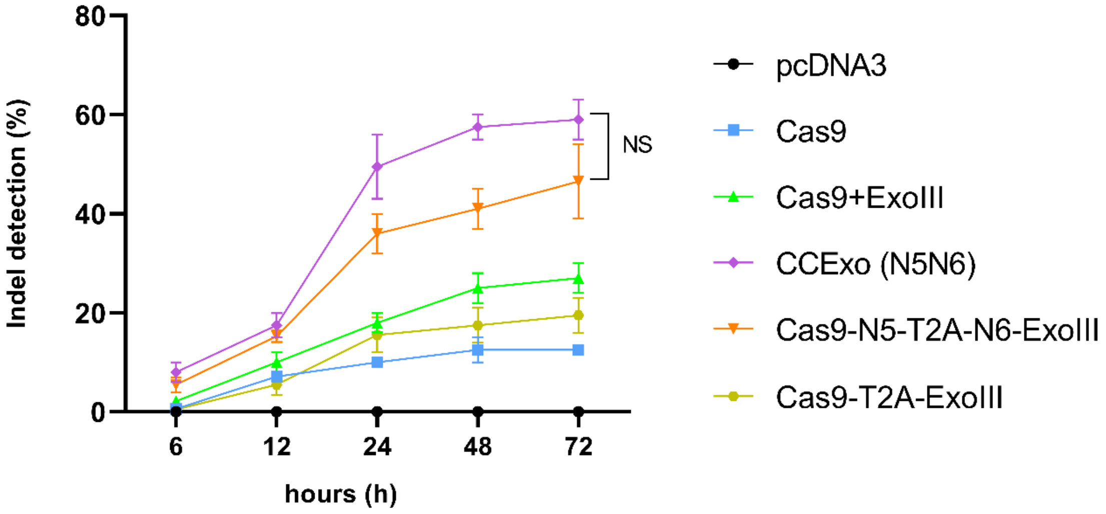

Supplementary Fig. 12: Kinetics of CCExo in cellulo. HEK293 cells ($2*10^5$ cells/ml) were transfected with different variants for enhancing genome editing with CRISPR/Cas system targeting *hVEGFα* gene. At the indicated time, genomic DNA was isolated, target genomic region was PCR amplified and later T7E1 assay for indel detection was performed. Data present three individual separate experiments (n=3) with ordinary one-way ANOVA followed by Tukey's multiple comparisons test. Data are presented as mean values +/- SEM as appropriate.

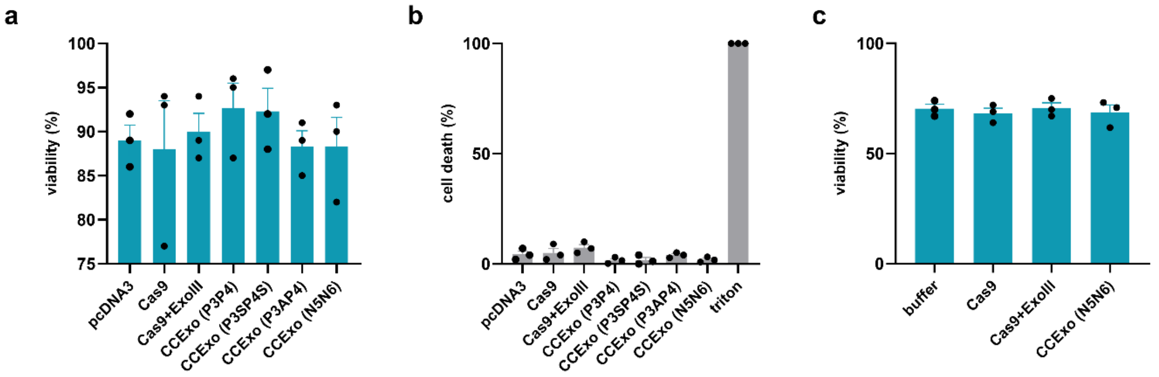

Supplementary Fig. 13: Cell viability after CCExo expression in human cells. HEK293 cells (2*10^5 cells/ml) were transfected with different variants for enhancing genome editing with CRISPR/Cas system. 48 hours later, cells were stained with trypan blue to determine the cell viability (a). LDH release was determined in cell media (b). Human $CD3^+$ cells (1x10^7 cells/ml) were electroporated with various proteins (300nM of Cas9 or variants and exonucleases) and 48 hours later cell viability was tested with trypan blue staining (**c**). Data present three individual separate experiments (n=3). Data are presented as mean values +/- SEM as appropriate.

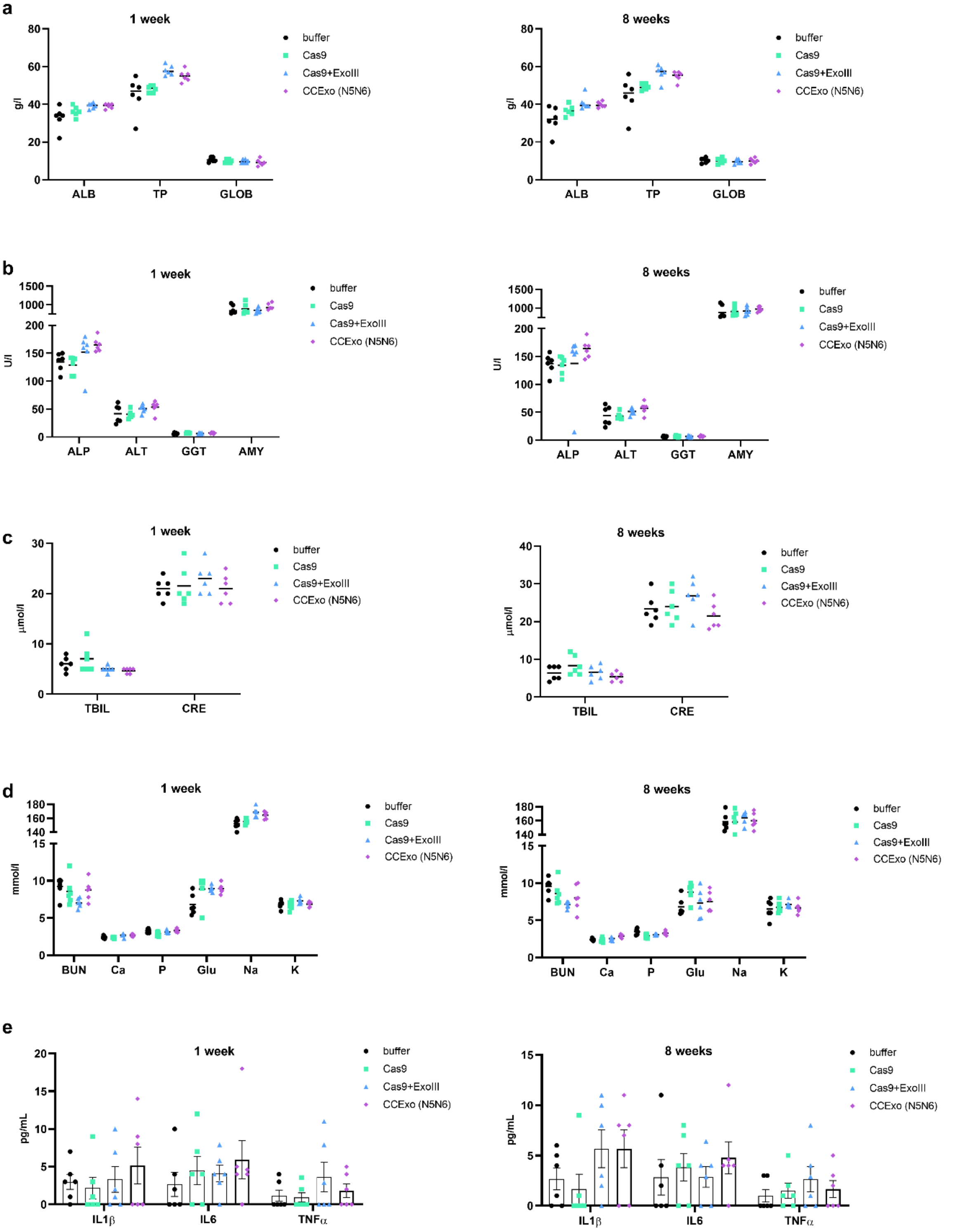

Supplementary Fig. 14: Testing the toxicity of CCExo components in animals. Balb/c mice (n=6) were intravenously injected with 100 µg of Cas9 or Cas9-N5 protein and 100 µg of ExoIII or N6-ExoIII protein. After first and eighth weeks, blood was drawn and complete

biochemical panel (a-d) and proinflammatory cytokines were screened (e). Data are presented as mean values +/- SD as appropriate.

Abbreviations: ALB, albumins; TP, total proteins; GLOB, globulins; ALP, alkaline phosphatase; ALT, alanine aminotransferase; GGT, gamma glutamyl transferase; AMY, amylase; TBIL, total bilirubin; CRE, creatinine kinase; BUN, blood urea nitrogen; Glu, glucose.

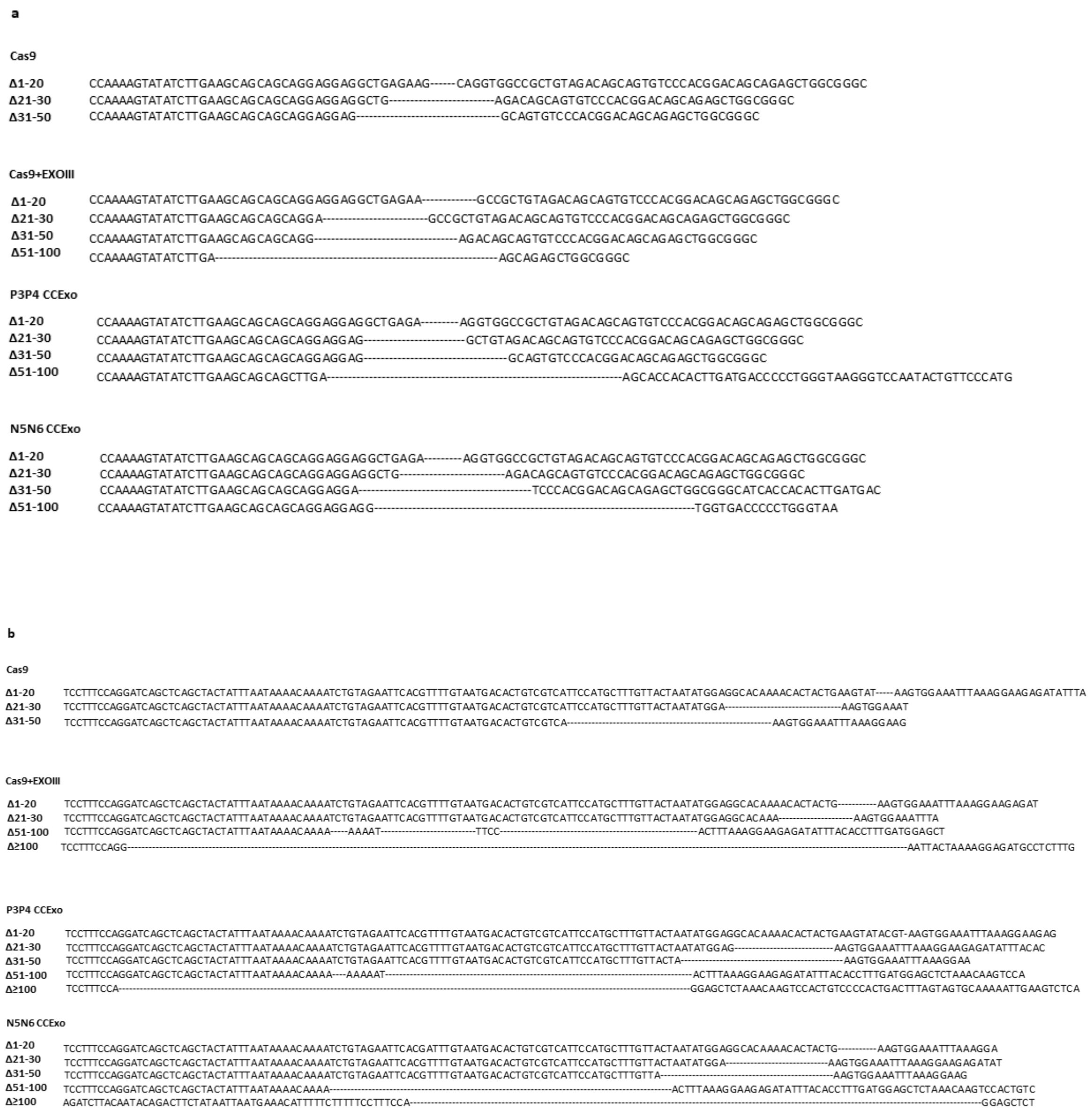

Supplementary Fig. 15: NGS results of *MYD88 (a; 200 bp amplicons) and CD47 (b; 400 bp amplicons)* gRNA targeting PCR amplicon. Extended data related to figure 3. Representative sequences for each deletion lengths are presented.

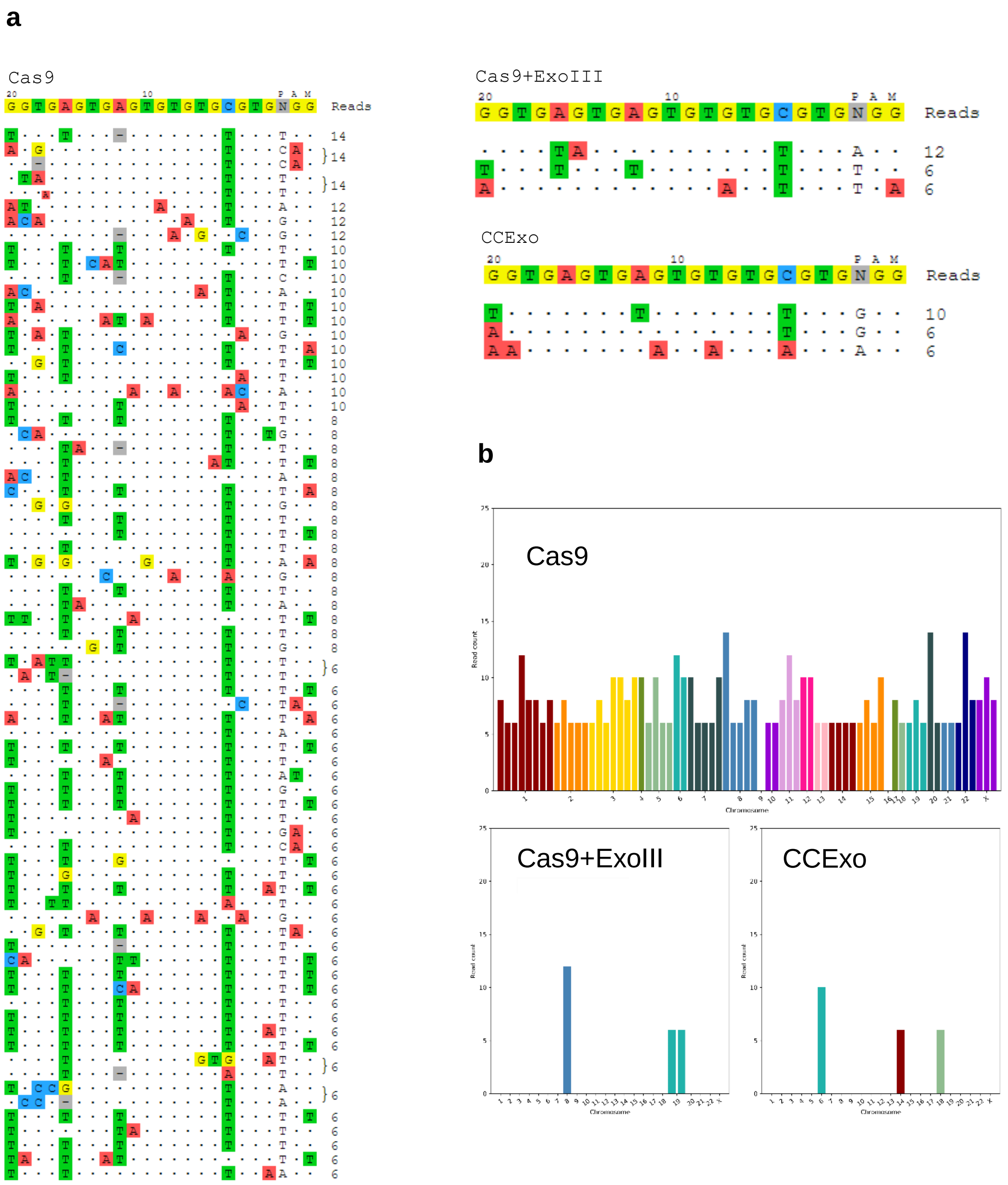

Supplementary Fig. 16: Visualization of off-target sites detected by CIRCLE-seq analysis for *VEGFα* site in HEK cells. The intended target site sequence is placed on the top. Off-target sites are put in order by CIRCLE-seq read (identified matched read compared to target sequence), where mismatches to the intended target sequence is visualized using colored nucleotides, whereas matches are denoted with dots (**a**). Manhattan plots of CIRCLE-seq detected off-target sites with bar heights representing read count and organized by chromosomal position (**b**).

a

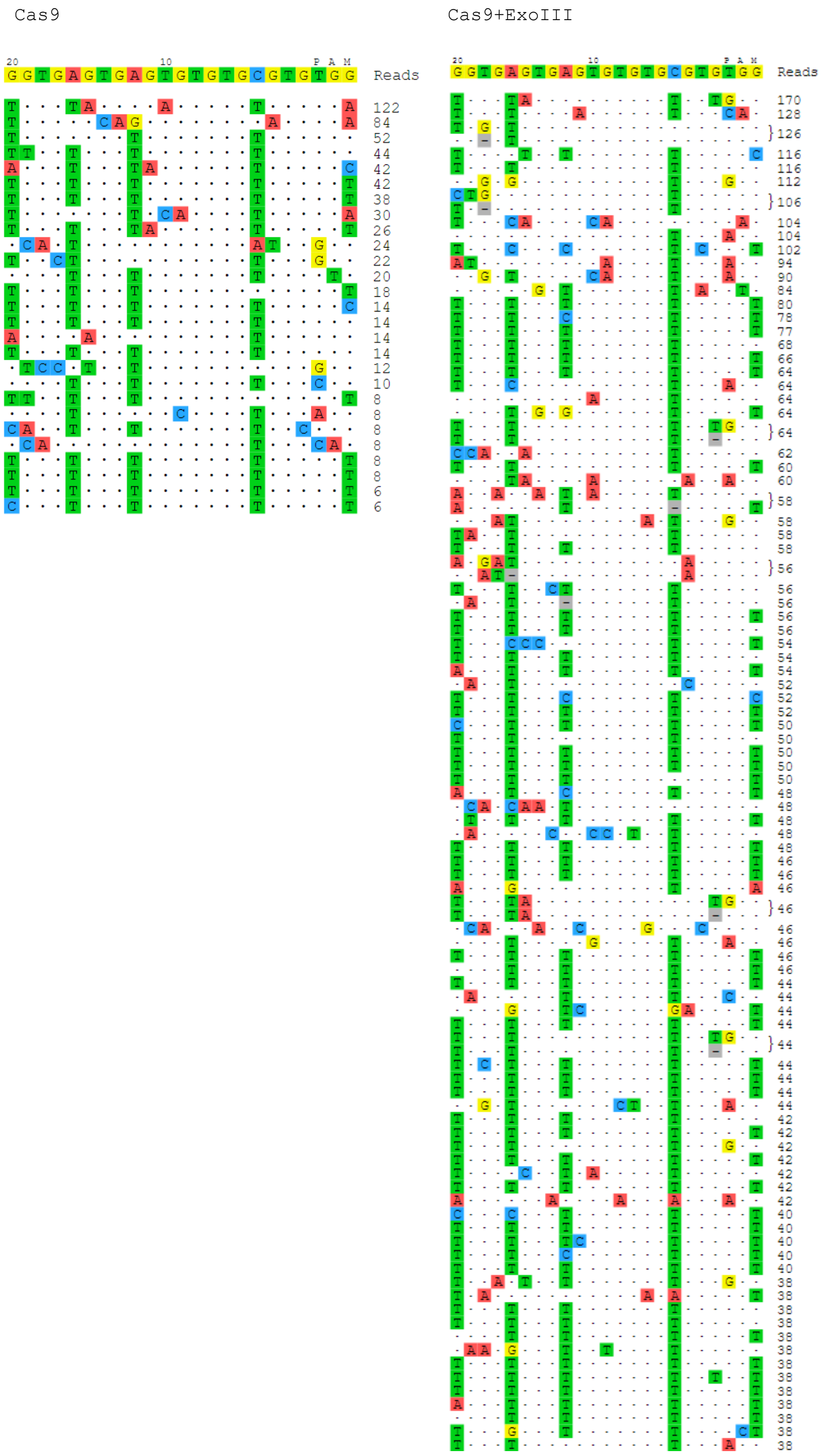

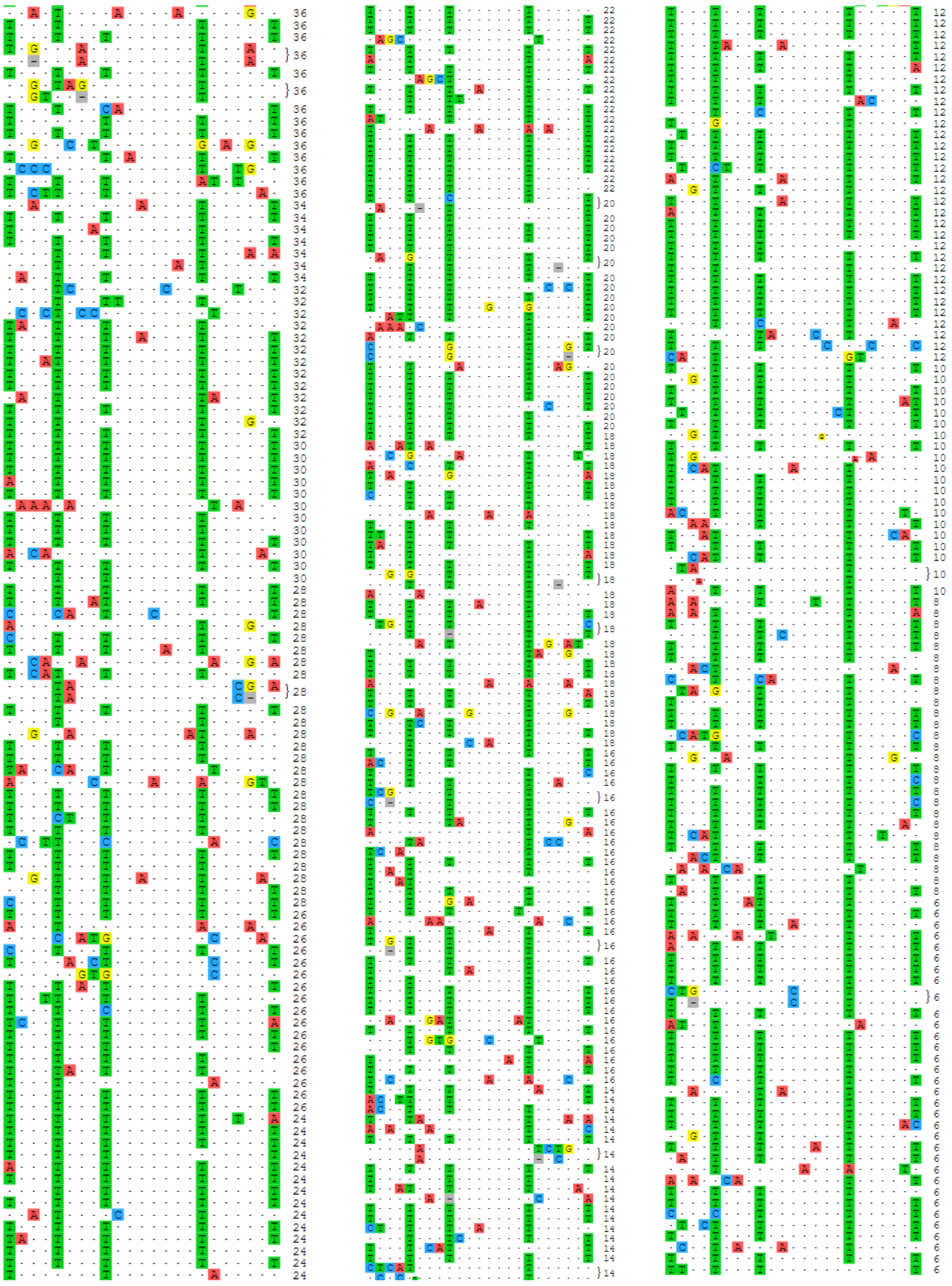

CCExo

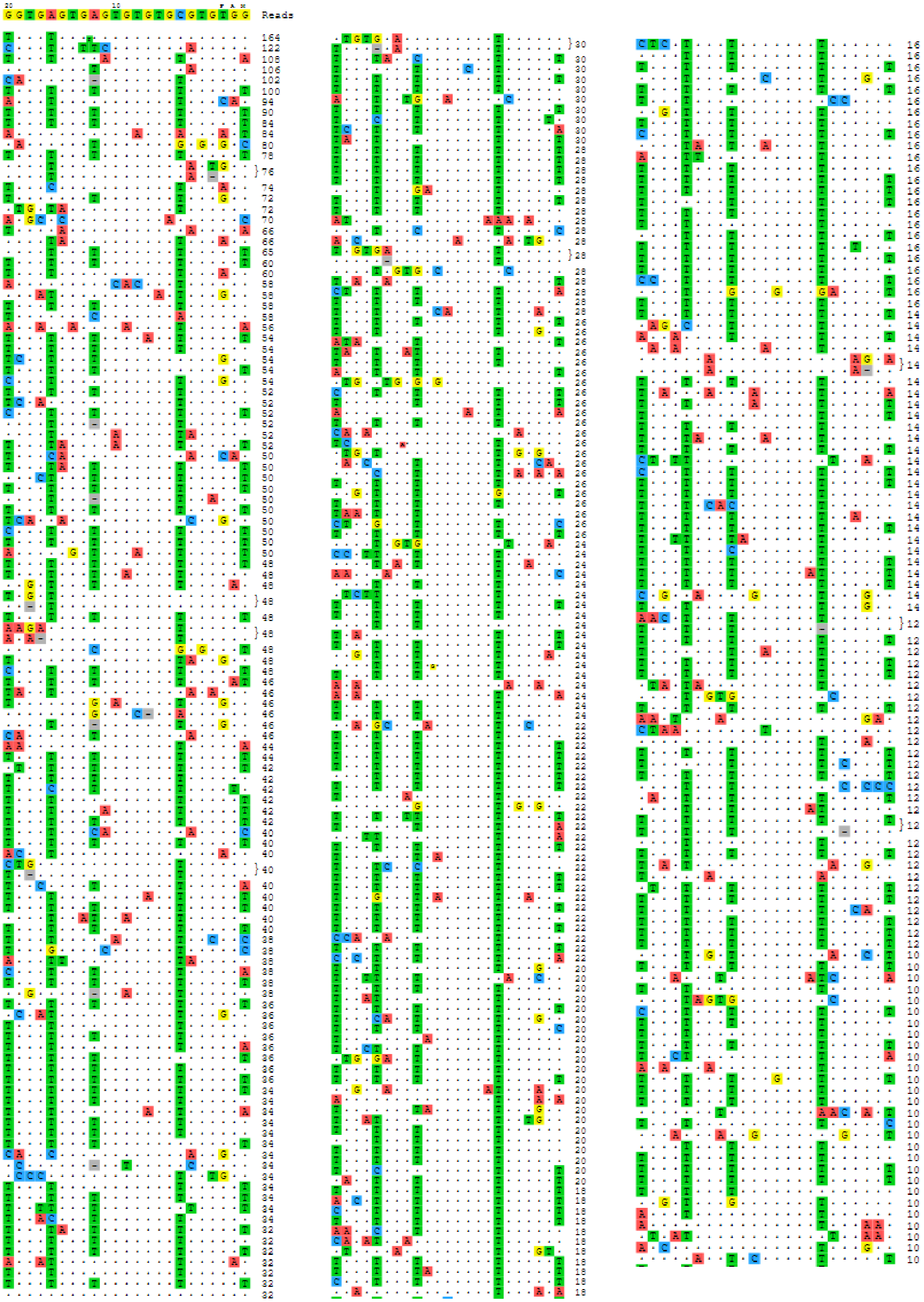

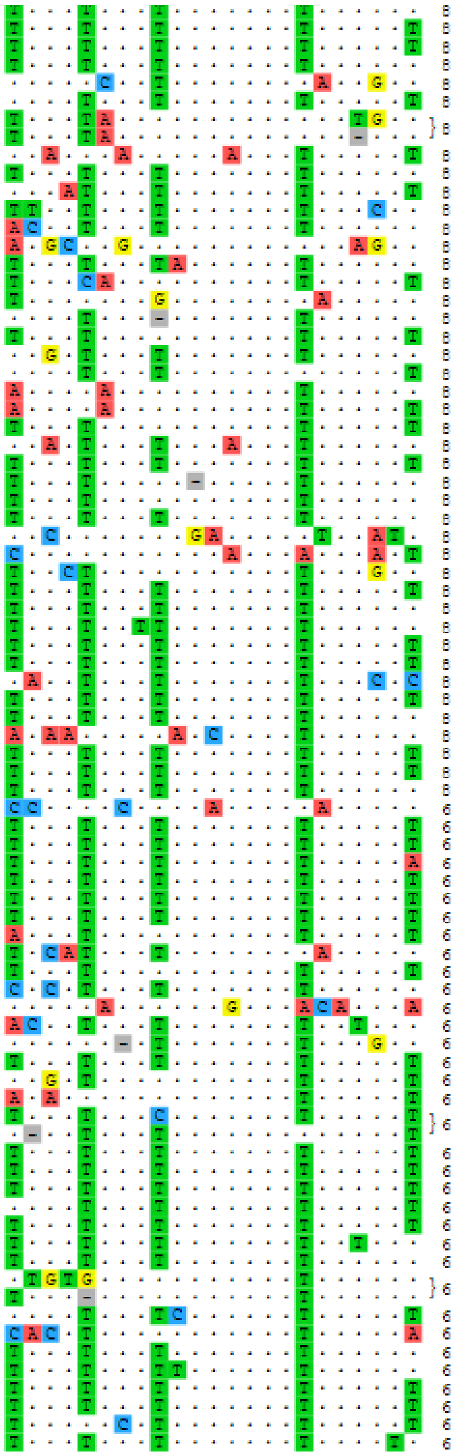

b

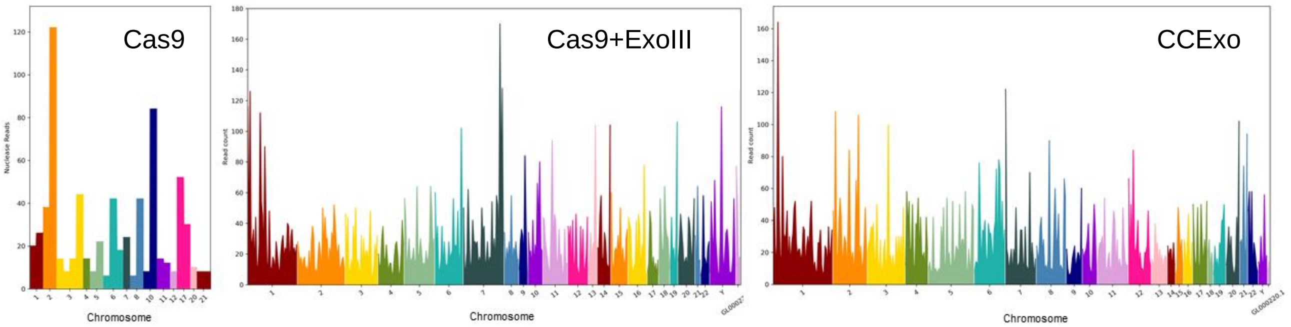

Supplementary Fig. 17: Visualization of off-target sites detected by CIRCLE-seq analysis for *VEGFα* site in K562 cells. The intended target site sequence is placed on the top. Off-target sites are put in order by CIRCLE-seq read (identified matched read compared to target sequence), where mismatches to the intended target sequence is visualized using colored nucleotides, whereas matches are denoted with dots (**a**). Manhattan plots of CIRCLE-seq detected off-target sites with bar heights representing read count and organized by chromosomal position (**b**)

a

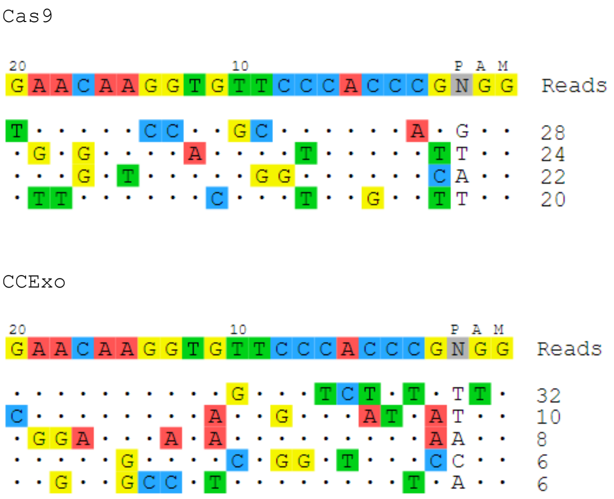

b

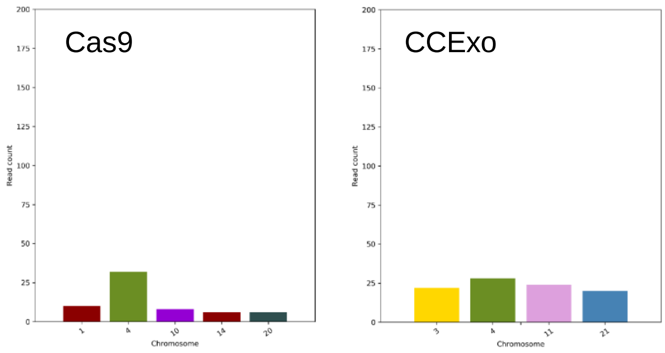

Supplementary Fig. 18: Visualization of off-target sites detected by CIRCLE-seq analysis for *TRBC1* site in K562 cells. The intended target site sequence is placed on the top. Off-target sites are put in order by CIRCLE-seq read (identified matched read compared to target sequence), where mismatches to the intended target sequence is visualized using colored nucleotides, whereas matches are denoted with dots (**a**). Manhattan plots of CIRCLE-seq detected off-target sites with bar heights representing read count and organized by chromosomal position (**b**)

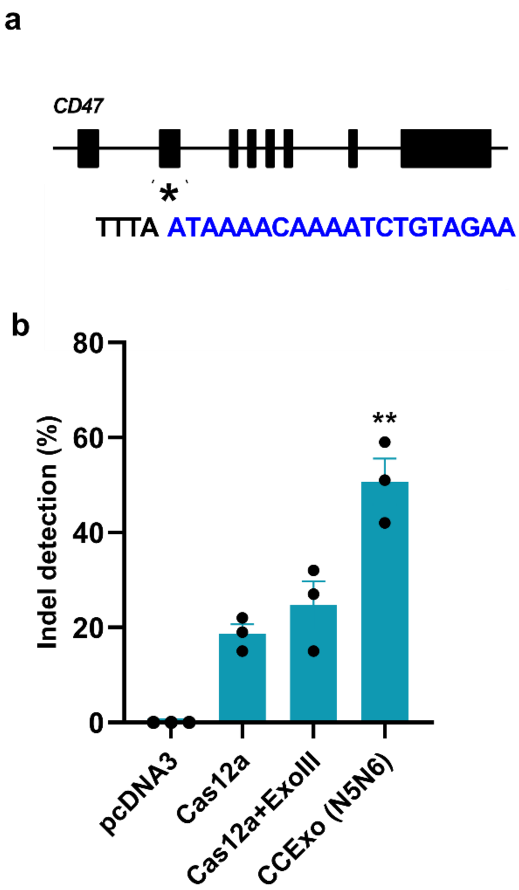

Supplementary Fig. 19: CCExo approach for enhancing Cas12a action. DNA sequence of human *CD47* gene for designing gRNA (blue-seeding sequence, black-PAM, * denote the site of editing within the gene) for genome editing (a). HEK293 cells (2*10^5 cells/ml) were cotransfected with plasmids for Cas12a, gRNA or CCExo (600 ng of each component). 48 hours later genomic DNA was isolated, target genomic region was PCR amplified and later T7E1 assay for indel detection was performed (b). Data present three individual separate experiments (n=3). **P< 0.0001. All P values are from ordinary one-way ANOVA followed by Tukey's multiple comparisons test compared to Cas12a values. Data are presented as mean values +/- SEM as appropriate.

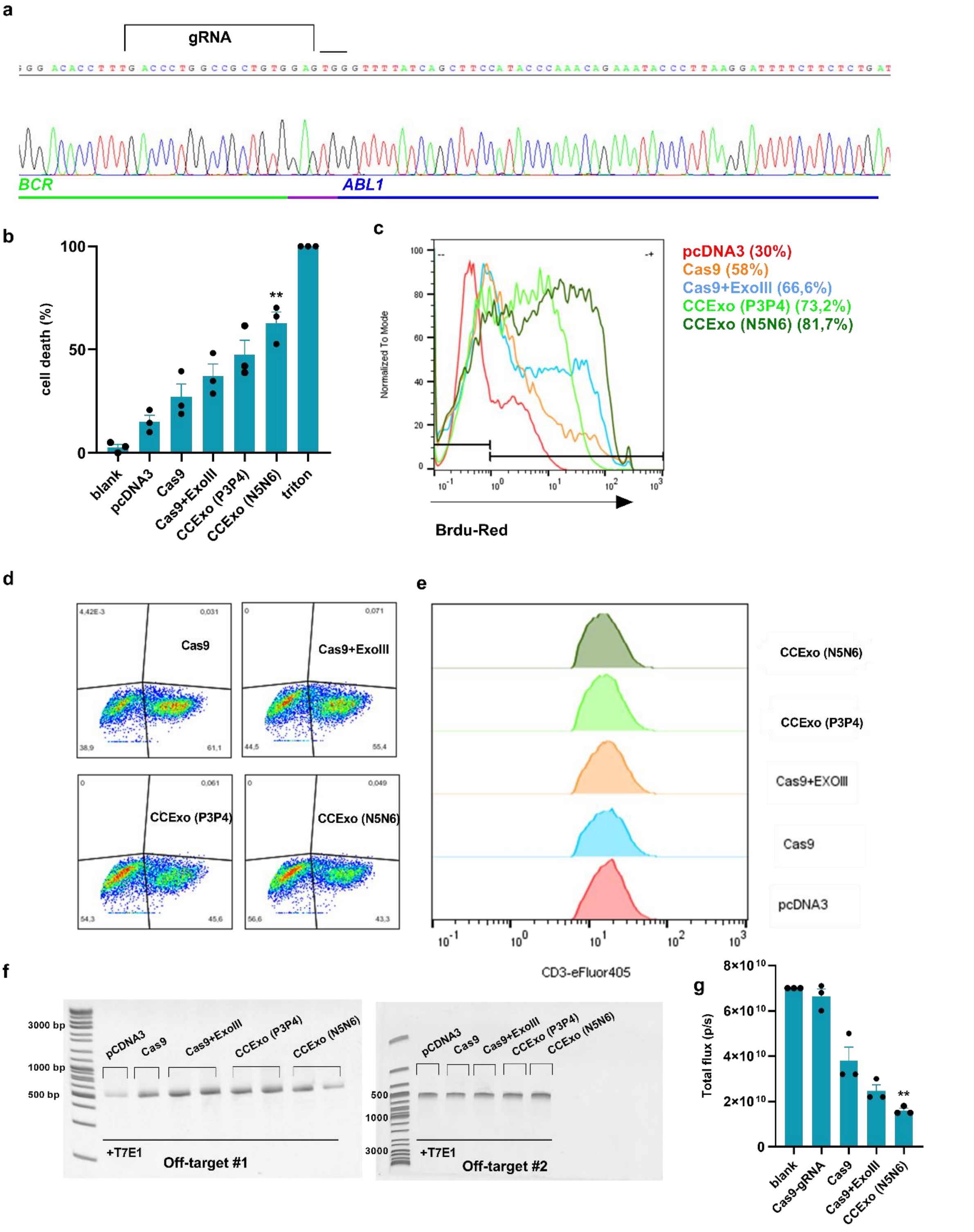

Supplementary Fig. 20: Philadelphia chromosome gene inactivation in K562 cells by CCExo system. Sanger sequencing of K562 cell line showing *BCR*-*ABL1* fusion break point (green-*BCR*, blue-*ABL1*, violet-microhomology region) upon which gRNA was designed. Bracket denotes the gRNA target sequence used for further editing of the Philadelphia

chromosome (a). K562 cells were electroporated with depicted plasmids, where gRNA was targeting *BCR-ABL1* junction site. Cell death was determined by measuring LDH release in cell media 48 hours after electroporation. Data present three individual separate experiments (n=3). **P< 0.01. All P values are from ordinary one-way ANOVA followed by Tukey's multiple comparisons test compared to Cas9 values. Data are presented as mean values +/- SEM as appropriate (b). Cell death of K562 cells after gene editing(electroporation with plasmids), additionally confirmed by TUNEL assay (c). Co-culture of K562-fLUC-GFP (1,5*10^6 cells) and Jurkat cells (1,5*10^6 cells) were electroporated with denoted plasmids. Loss of GFP due (right lower quadrant) to cell death was determined by FACS analysis (d). Jurkat cells within treated co-culture (as stated in panel d) were stained against CD3. Histograms of CD3 percentage of FACS analysis showing specificity of CCExo induced cell death only in K562-fLUC-GFP cells (e). T7E1 assay for indel detection of predicted off-target sites was performed. A representative image from two individual separate experiments is shown (f). *De novo* isolated recombinant proteins form RNPs were used for K562-fLUC electroporation to test specific gene editing. Cell death correlates with drop of bioluminescence values. Data present three individual separate experiment (n=3). **P< 0.01. All P values are from ordinary one-way ANOVA followed by Tukey's multiple comparisons test compared to Cas9 values. Data are presented as mean values +/- SEM as appropriate. (g).

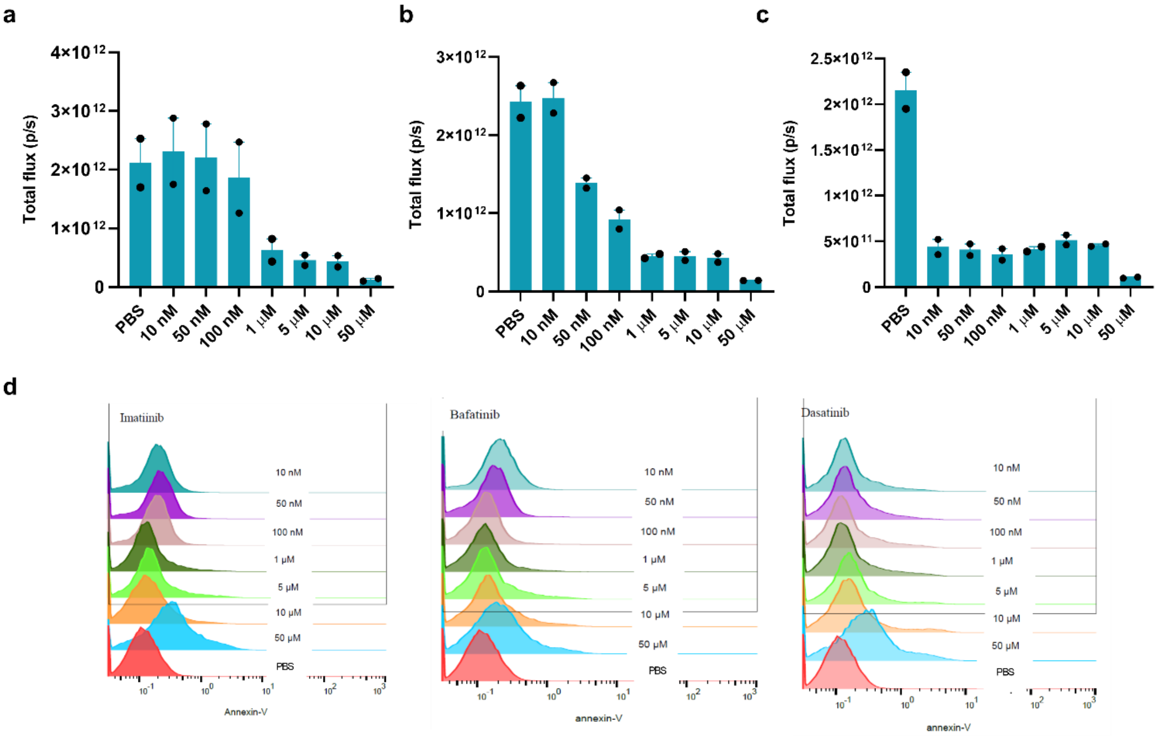

Supplementary Fig. 21: Inhibition of specific kinase receptor of *BCR-ABL1* fusion cell model, K562 cells. K562-fLUC cells (10^6 cells/ml) were treated with various doses of inhibitor and afterwards (24h later) cell death by measuring bioluminescence was determined; treatment with imatinib (a), treatment with bafatinib (b) and treatment with dasatinib (c). Data present two individual separate experiments (n=2). Data are presented as mean values +/- SEM as appropriate. Cell death was additionally determined by annnexin-V staining (d).

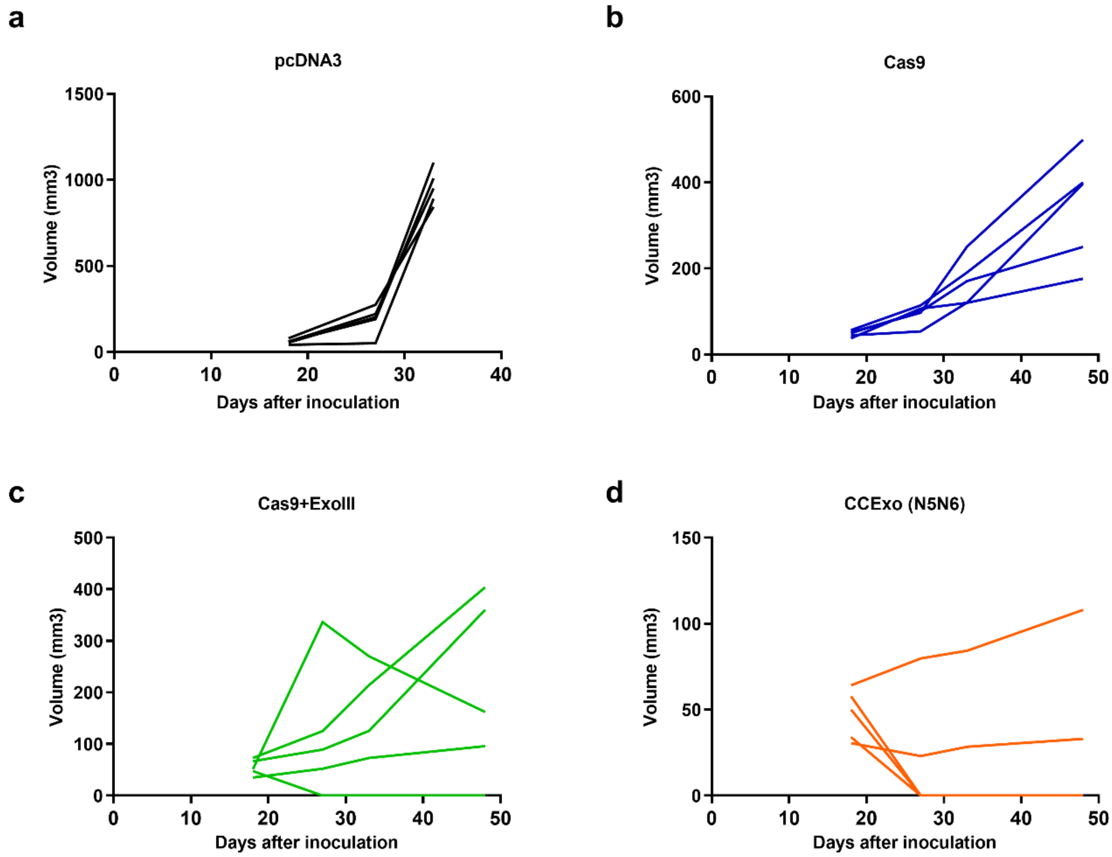

Supplementary Fig. 22: CCExo for possible therapeutic use. Tumor volume from animals in which K562-xenograft tumor was electroporated with pcDNA3 plasmid (a), Cas9 plasmid (b), plasmids, expressing Cas9 and *E.coli* ExoIII (c) and plasmids expressing the CCExo (N5N6) system (d). Each line represents growth of a tumor within a single animal.

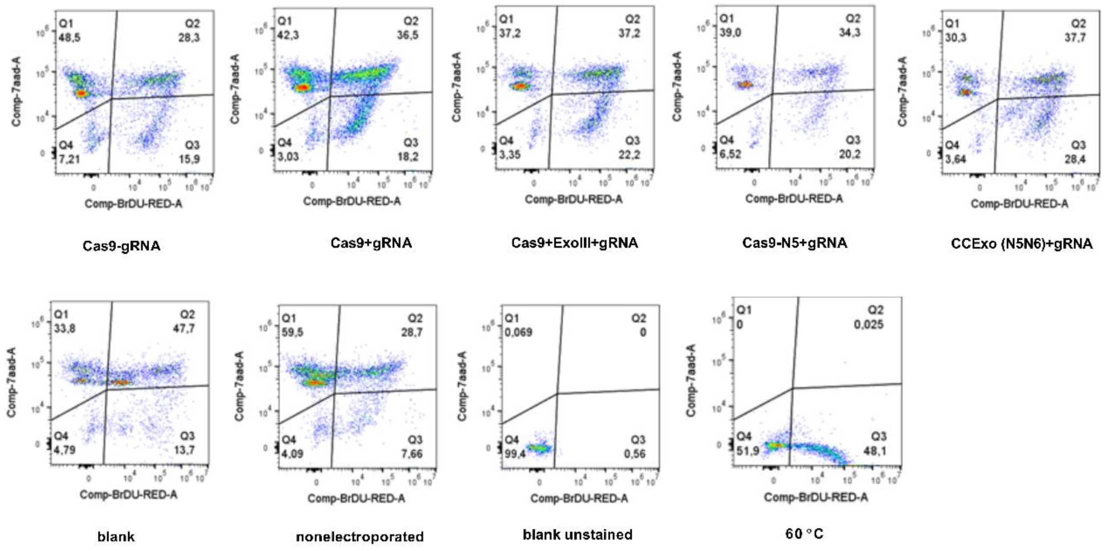

Supplementary Fig. 23: FACS analysis of TUNEL assay on CML patient cells. PBMCs (10^7cells/ml) of CML patients were electroporated with Cas9 or CCExo RNPs, whereas cells, heated at 60°C were used as test positive control. 72 hours later, cells were subjected to TUNEL assay with subsequent FACS analysis. Brdu-Red positive cells (right lower quadrant) showing elevated cell death when CCExo RNPs were used. Representative analysis for data related to figure 4d.

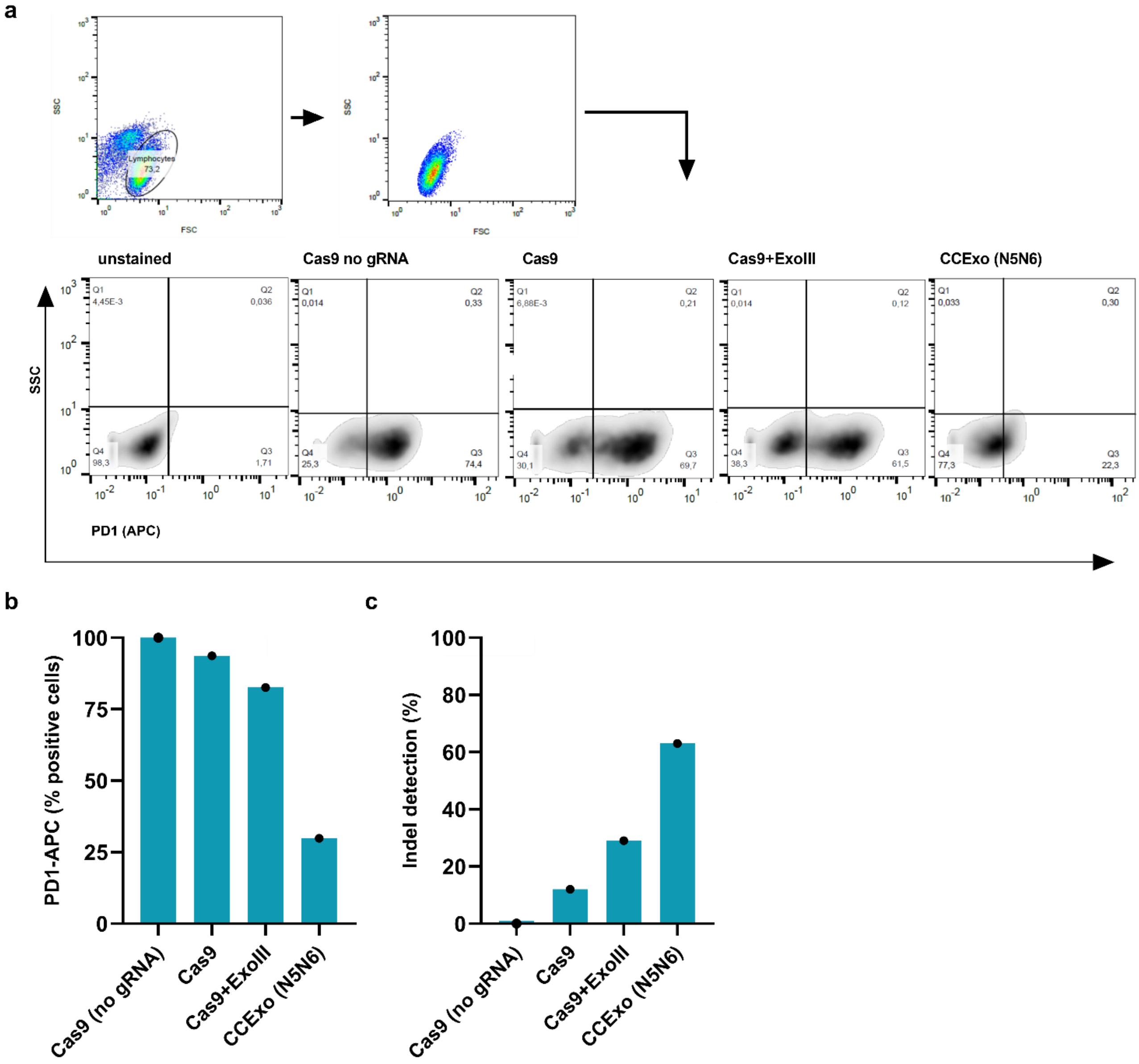

Supplementary Fig. 24: CCExo for inactivation of a PD-1 checkpoint mediator for T cell engineering. PD1-APC staining showing CCExo RNP mediated PD1 gene disruption on T cells. Gating strategy is also shown. (a). PD1 gene disruption death was determined four days after the RNPS electroporation by FACS analysis, where % of PD1 positive cell of Cas9 (no gRNA) electroporation condition were taken as 100%. (b). T7E1 assay for indel detection of predicted off-target sites was performed four days after the RNPs were electroporated in bulk T cells (c).

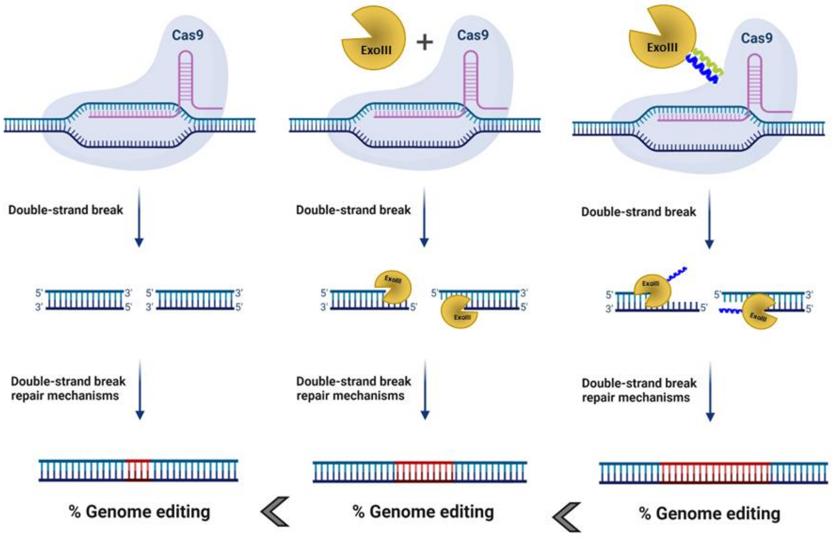

Supplementary Fig. 25: Schematic presentation of the principle of enhanced gene inactivation via the CCExo system. Due to the presence of an exonuclease, which enzymatically degrades free 3' regions of Cas9-cleaved DNA, larger recessions are formed at the cleavage site, which lead to higher efficiencies of genome editing, compared to editing with Cas9 alone. Efficiency is further improved via coiled-coil tethering of the two enzymes through dynamic coiled-coil based interactions positioning Cas9 and the exonuclease in sufficient proximity at the target site, allowing maximal efficiency of the CCExo system.

Supplementary Table 1. gRNA target sequences in this study.

gene	sgRNA target sequences	Origin of target sequence
***eGFP* sgRNA**	GGCGAGGGCGATGCCACCTA CGG	This study
***CASP3* sgRNA**	AGAATAATTTGTACCCCGCT GGG	This study
***TRAC* sgRNA**	TGTGCTAGACATGAGGTCTA AGG	Stadtmauer EA. et al. Sicence, 2020
***mPtgs1* sgRNA**	CACAGCTGTGCTGCTGCCCA TGG	This study
***TLR4* sgRNA**	GAAGCAACATCTATCTGAAG AGG	This study
***ABL1* sgRNA**	ATCACTGAGTTCATGACCTA CGG	This study
***CD47* Cas12a sgRNA**	TTTA ATAAAACAAAATCTGTAGAA	This study
***PDCD1* sgRNA**	GGCGCCCTGGCCAGTCGTCT TGG	Stadtmauer EA. et al. Sicence, 2020
***VEGFα* sgRNA**	GGTGAGTGAGTGTGTGCGTG TGG	Fu Y. et al. NaBiotech, 2013
***EMX1* sgRNA**	GAGTCCGAGCAGAAGAAGAA GGG	Fu Y. et al. NaBiotech, 2013
***MYD88* sgRNA**	GGCTGAGAAGCCTTTACAGG TGG	This study
***CD47* sgRNA1**	ATGCTTTGTTACTAATATGG AGG	This study
***CD47* sgRNA2**	AGAGATATTTACACCTTTGA TGG	This study
***CD47* sgRNA3**	CTACTGAAGTATACGTAAAG TGG	This study
***CD47* sgRNA4**	AGGAGATGCCTCTTTGAAGA TGG	This study
***CD47* sgRNA5**	TCCATATTAGTAACAAAGCA AGG	This study
***CD47* sgRNA6**	ACTAAAGTCAGTGGGGACAG TGG	This study
***BCR-ABL1* sgRNA**	GACCCTGGCCGCTGTGGAG TGG	This study
***TRBC1* sgRNA**	GAACAAGGTGTTCCCACCCG AGG	This study

Supplementary Table 2. Amino acid sequences of coiled-coiled forming peptides

name	AA sequence
**P3**	EIQQLEE EIAQLEQ KNAALKE KNQALKY
**P4**	KIAQLKQ KIAQLKQ ENQQLEE ENAALEY
**N5**	EIAALEA KIAALKA KNAALKA EIAALEA
**N6**	KIAALKA EIAALEA ENAALEA KIAALKA
**P3S**	EIQQLEE EISQLEQ KNSQLKE KNQQLKY
**P4S**	KISQLKQ KIQQLKQ ENQQLEE ENSQLEY
**AP4**	ELAANEE ELQQNEQ KLAQIKQ KLQAIKY

Supplementary Table 3. Primer sequences for CIRCLE-seq predicted Off-target determination

Name	Primer forward	Primer reverse
**VEGF on-target**	TGTTTGGGAGGTCAGAAATAGGGGGTC	AGGGAGCAGGAAAGTGAGGT
**VEGF OT1 (off-target)**	CCTGCTCAGCACCTGCACTTCT	TGGCATTCTCCTGGGTGTGGTG
**VEGF OT2**	GGGTTACATGACTTTGTTTTCAATGGCT	CACAGAAGGATGTGTGCAGGT
**VEGF OT3**	GACAGAACATCGGAAGGGGCCC	CCTGCCTTGCCATCCTCCTCTT
**VEGF OT4**	TCTGACCACAGAAGTGGTTGATG	CTTCAATTTTTCTTCTTGAGAGGGTT
**VEGF OT5**	TATCCTGATGTGATTATGATGTATTA	TAGCAGGTATATATATGTAATTTTTG
**VEGF OT6**	AGAGAGAGACTGAGAGAAAAAC	TCTCTCAACGTCTCTCTCTCTCTC
**VEGF OT7**	GCTCAGGGGGTCGTGGAAGAAG	TTTCCACTCTGTCCCCACGTCT
**VEGF OT8**	ACGTGGTACTGCGCTTTGCTCT	AGGTACAGTCTGTGAGCTCTGA
**TRBC1 on-target**	TCTCAGAGCAACCCTGGCTCCAAC	CTGGACTTGACAGCGGAAGTGGTTG
**TRBC1 OT1**	CAGAGAATCAATCTCTCTTTTATC	TCACTGAGGAAGCCTCATCCAC
**TRBC1 OT2**	CCACTTGCTGTGTGGGGTCTAGAC	TACGCAGTTGTTTGACAGGCAT
**TRBC1 OT3**	GTTGAAGATAACTGGTTGTCTTC	ACTAGCTCCTGCCCACACTCTG
**TRBC1 OT4**	CGCGGGAGAAGAGGGAAAGAGC	GGCACCTTCTCCATGTAGCCCC
