# Supplementary material for: Coiled-coil heterodimer-based recruitment of an exonuclease to CRISPR/Cas for enhanced gene editing
Source: Nat Commun. 2022 Jun 23;13:3604. doi: 10.1038/s41467-022-31386-1 (PMC9226073; doi:10.1038/s41467-022-31386-1)
Supplement: Supplementary file 2 — Reporting Summary [file 41467_2022_31386_MOESM2_ESM.pdf]

nature portfolio

Corresponding author(s): prof.dr.Roman Jerala
Last updated by author(s): Jun 13, 2022

Reporting Summary

Nature Portfolio wishes to improve the reproducibility of the work that we publish. This form provides structure for consistency and transparency in reporting. For further information on Nature Portfolio policies, see our Editorial Policies and the Editorial Policy Checklist.

Statistics

For all statistical analyses, confirm that the following items are present in the figure legend, table legend, main text, or Methods section.

n/a	Confirmed	
☐	☒	The exact sample size (*n*) for each experimental group/condition, given as a discrete number and unit of measurement
☐	☒	A statement on whether measurements were taken from distinct samples or whether the same sample was measured repeatedly
☐	☒	The statistical test(s) used AND whether they are one- or two-sided<br>*Only common tests should be described solely by name; describe more complex techniques in the Methods section.*
☐	☒	A description of all covariates tested
☐	☒	A description of any assumptions or corrections, such as tests of normality and adjustment for multiple comparisons
☐	☒	A full description of the statistical parameters including central tendency (e.g. means) or other basic estimates (e.g. regression coefficient) AND variation (e.g. standard deviation) or associated estimates of uncertainty (e.g. confidence intervals)
☐	☒	For null hypothesis testing, the test statistic (e.g. *F*, *t*, *r*) with confidence intervals, effect sizes, degrees of freedom and *P* value noted<br>*Give P values as exact values whenever suitable.*
☒	☐	For Bayesian analysis, information on the choice of priors and Markov chain Monte Carlo settings
☒	☐	For hierarchical and complex designs, identification of the appropriate level for tests and full reporting of outcomes
☒	☐	Estimates of effect sizes (e.g. Cohen's *d*, Pearson's *r*), indicating how they were calculated

Our web collection on statistics for biologists contains articles on many of the points above.

Software and code

Policy information about availability of computer code

Data collection	clone https://github.com/tsailabSJ/circleseq.git for CIRCLE-seq analysis of NGS obtained sequences, hg38 for CIRCLE-seq analysis
Data analysis	clone https://github.com/tsailabSJ/circleseq.git and hg38 for CIRCLE-seq analysis of NGS obtained sequences, Phyton v2.7, BWA 0.7.17 and SAMtools 1.9 for CIRCLE-seq data analysis, Phyton 3.7 and Matplotlib 3.5 for Manhattan plot visualization, CRISPResso or Cas-analyzer for NGS amplicon analysis, ICE Synthego and TIDE on-line tool for Sanger sequences analysis, FlowJo v10 and SpectroFlo for flow-cytometry analysis, Living Image® 4.5.2 (Perkin Elmer) for bioluminiscence imaging quantification, Leica LAS AF Lite version 2.7.2.9586 for confocal images, ImageJ 1.53k for gel density analysis, Excel 2010 and Microsof PowerPoint 2010, GraphPad Prism8, Biorender.com for schemes drawing.

For manuscripts utilizing custom algorithms or software that are central to the research but not yet described in published literature, software must be made available to editors and reviewers. We strongly encourage code deposition in a community repository (e.g. GitHub). See the Nature Portfolio guidelines for submitting code & software for further information.

Data

Policy information about availability of data

All manuscripts must include a data availability statement. This statement should provide the following information, where applicable:

- Accession codes, unique identifiers, or web links for publicly available datasets
- A description of any restrictions on data availability
- For clinical datasets or third party data, please ensure that the statement adheres to our policy

Exact P values from all analysis, CIRCLE-seq identified matches are provided in source Data file. All other data are available from the authors of the paper upon request. Data from NGS and CIRCLE-seq sequencing are deposited at NCBI SRA with accession number PRJNA849175.

Field-specific reporting

Please select the one below that is the best fit for your research. If you are not sure, read the appropriate sections before making your selection.

[x] Life sciences [ ] Behavioural & social sciences [ ] Ecological, evolutionary & environmental sciences

For a reference copy of the document with all sections, see nature.com/documents/nr-reporting-summary-flat.pdf

Life sciences study design

All studies must disclose on these points even when the disclosure is negative.

Sample size	We did not use any sample, size calculation; the sample size was chosen based on the published literature and Sample size was chosen depending on the technique used and based on our experience (Methods/Quantification and Statistical Analysis). No power calculations were performed to choose group size
Data exclusions	No data was excluded from the analysis.
Replication	At least three independent replicated experiments were performed except for the animal experiments (n=5), NGS analysis and PD1, where one replicate experiment were done. We used two different blood donors from CML patients. All attempts of replication were successful.
Randomization	In analysis, where cells were used, three independent measurements are presented. No randomization was carried out. In animal studies animals were randomly distributed between experimental groups.
Blinding	Blinding was performed in animal research. In tumor measurements, the person, who measured tumors was not aware of the group type. Otherwise no blinding was performed in other experiments.

Reporting for specific materials, systems and methods

We require information from authors about some types of materials, experimental systems and methods used in many studies. Here, indicate whether each material, system or method listed is relevant to your study. If you are not sure if a list item applies to your research, read the appropriate section before selecting a response.

Materials & experimental systems

n/a	Involved in the study	
[ ]	[x]	Antibodies
[ ]	[x]	Eukaryotic cell lines
[x]	[ ]	Palaeontology and archaeology
[ ]	[x]	Animals and other organisms
[ ]	[x]	Human research participants
[x]	[ ]	Clinical data
[x]	[ ]	Dual use research of concern

Methods

n/a	Involved in the study	
[x]	[ ]	ChIP-seq
[ ]	[x]	Flow cytometry
[x]	[ ]	MRI-based neuroimaging

Antibodies

Antibodies used	mouse anti-CRISPR-Cas9 antibody (ab191468) from Abcam; 1:500<br>rabbit anti-Alpha/Beta tubulin antibody (2148) from Cell Signaling Technology; 1:2000<br>goat polyclonal anti-rabbit IgG (ab6721) from Abcam; 1:2000<br>goat anti-mouse IgG-HRP (sc-2005) from Santa Cruz; 1:2000<br>Alexa Fluor® 488 goat anti-mouse IgG (H+L) *2 mg/mL from Invitrogen (A11001); 1:2000<br>CD3 Antibody, anti-human, eFluor405 from Miltenyi Biotec (130-113-128); 1:50<br>CD279 (PD1)-PE-Vio770, human from Miltenyi Biotec (130-117-698); 1:50<br>CD47-FITC, human from Miltenyi Biotec (130-101-344); 1:50
Validation	All antibodies used in the study are commercially available and have been validated according to manufacturers manuals.

Eukaryotic cell lines

Policy information about cell lines

Cell line source(s)	human embryonic kidney (HEK) 293, mouse Neuro-2A, K562 cells and Jurkat cells were purchased from American Type Culture Collection (ATCC). The 293/GFP cell line, which stably expresses GFP, was purchased from Cell Biolabs. K562-fLUC

	cells were a kind gift from Sebastien Wälchli (they generated the cells that stably expresses firefly luciferase). CML patient cells and PBMCs for T cell isolation were obtained from human volunteers upon their consent.
Authentication	None were authenticated.
Mycoplasma contamination	All cells were Mycoplasma negative.
Commonly misidentified lines (See ICLAC register)	No commonly misidentified cell lines were used in the study

Animals and other organisms

Policy information about studies involving animals; ARRIVE guidelines recommended for reporting animal research

Laboratory animals	female 8-10 weeks SCID C.B-478 17/IcrHsd-Prkdcscid mice (Envigo, Italy)<br>female 8-10 weeks Hsd:ICR (CD-1) (Envigo, Italy)<br>female 8-10 weeks BALB/c OlaHSd (Envigo, Italy)<br>female 8-10 weeks B6(C)-Gt(ROSA)26Sorem1.1(CAG-cas9*,-EGFP)Rsky/J (JAX, USA)<br>Laboratory animals were housed in IVC cages (Techniplast), fed standard chow (Mucedola) and tap water was provided ad libitum. Mice were maintained in 12-12 hour dark-light cycle at approximately 40-60% relative humidity with 22°C of ambient temperature. All animals, used in the study, were healthy; accompanied with health certificate from the animal vendor.
Wild animals	No wild animals were used in the study.
Field-collected samples	No field collected samples were used in the study.
Ethics oversight	Administration of the Republic of Slovenia for Food Safety, Veterinary and Plant Protection of the Ministry of Agriculture, Forestry and Foods, Republic of Slovenia (Permit Number U34401-3/2017/16, U34401-9/2020/9).

Note that full information on the approval of the study protocol must also be provided in the manuscript.

Human research participants

Policy information about studies involving human research participants

Population characteristics	Samples (blood for PBMC isolation) were obtained with informed consent, and according to the study protocol approved by National Medical Ethics Committee (0120-600/2019/3). Blood was drawn regardless the age and gender of the volunteer. The main criteria was that they are healthy.<br>Blood of CML patients for PBMC isolation was taken also from volunteers, regardless of the gender and age, the main criteria was that they did not get the CML therapy yet.
Recruitment	No specific recruitment of the participants was performed. The blood from the CML patients was drawn at the diagnosis of the disease.
Ethics oversight	National Medical Ethics Committee (permit number 0120-600/2019/3) of the Ministry of Health, Republic of Slovenia

Note that full information on the approval of the study protocol must also be provided in the manuscript.

Flow Cytometry

Plots

Confirm that:

- [x] The axis labels state the marker and fluorochrome used (e.g. CD4-FITC).
- [x] The axis scales are clearly visible. Include numbers along axes only for bottom left plot of group (a 'group' is an analysis of identical markers).
- [x] All plots are contour plots with outliers or pseudocolor plots.
- [x] A numerical value for number of cells or percentage (with statistics) is provided.

Methodology

Sample preparation	Cells were washed with FACS buffer (PBS, 2% FBS) and resuspended in a 0.1 ml FACS buffer and then stained with appropriate antibodies.
Instrument	CyFlow (Partec) or the spectral flow cytometer Aurora with the blue, violet and red lasers (Cytek)
Software	Data were analyzed with FlowJo software (Tree Star) and SpectroFlo (Cytek) software.
Cell population abundance	NA

Gating strategy

Cells were gated on live cell population, upon which cell were visualized for specific staining.

☒ Tick this box to confirm that a figure exemplifying the gating strategy is provided in the Supplementary Information.
